# Proteomic screening identifies HNRNPA2B1 as an epigenetic repressor of Epstein-Barr virus reactivation

**DOI:** 10.1128/jvi.00613-26

**Published:** 2026-06-12

**Authors:** Febri Gunawan Sugiokto, Yuxin Liu, Renfeng Li

**Affiliations:** 1 Program in Microbiology and Immunology, University of Pittsburgh 6614, Pittsburgh, Pennsylvania, USA; 2 Department of Microbiology and Molecular Genetics, University of Pittsburgh 6614, Pittsburgh, Pennsylvania, USA; 3 Cancer Virology Program, Hillman Cancer Center, University of Pittsburgh Medical Center 6595, Pittsburgh, Pennsylvania, USA; Dartmouth College Geisel School of Medicine, Hanover, New Hampshire, USA

**Keywords:** Epstein-Barr virus, HNRNPA2B1, latency, reactivation, Kaposi's sarcoma-associated herpesvirus, enChIP-MS, CRISPR, LSD1, H3K4Me3, RNA-binding proteins

## Abstract

Epstein-Barr virus (EBV) establishes lifelong persistent infection in over 90% of the world’s population. The virus persists as an episome in the host cells during latency and periodically reactivates through transcriptional activation of the immediate-early (IE) genes. While epigenetic regulation is central to maintaining viral latency, the host factors that enforce repression at these promoters remain incompletely defined. Here, we employed a novel Clustered Regularly Interspaced Short Palindromic Repeats (CRISPR)/Cas9-based engineered DNA-binding molecule-mediated chromatin immunoprecipitation coupled with mass spectrometry (enChIP-MS) approach to identify proteins associated with the promoter of EBV IE gene *ZTA*. This approach revealed an enrichment of multiple heterogeneous nuclear ribonucleoproteins and identified HNRNPA2B1 as a potential regulator of EBV *ZTA* gene expression. Functional analyses across multiple EBV+ cancer cell models demonstrated that HNRNPA2B1 acts as a restriction factor for EBV lytic reactivation. Depletion of HNRNPA2B1 led to increased expression of IE and downstream lytic genes, enhanced RNA polymerase II recruitment to the *ZTA* and *RTA* promoters, and elevated the proportion of cells entering the lytic cycle. Conversely, enforced expression of HNRNPA2B1 suppressed EBV lytic reactivation. Mechanistically, HNRNPA2B1 enhances repressive viral chromatin states by facilitating recruitment of the histone demethylase LSD1 to EBV IE gene promoters, thereby limiting the activating histone H3 lysine 4 trimethylation. Together, these findings identify HNRNPA2B1 as a key epigenetic regulator of EBV latency and link RNA-binding proteins to epigenetic control of viral reactivation.

**IMPORTANCE:**

This study identifies HNRNPA2B1 as a previously unrecognized host factor that promotes Epstein-Barr virus (EBV) latency through direct regulation of viral chromatin at immediate-early gene promoters. By integrating locus-specific chromatin proteomics with functional and mechanistic analyses, our work reveals how an RNA-binding protein HNRNPA2B1 recruits a histone-modifying enzyme to control EBV reactivation. These findings provide new insights into host-virus interactions that control EBV latency and reactivation and highlight the role of RNA-binding proteins in chromatin regulation that may be broadly relevant to other latent DNA viruses.

## INTRODUCTION

Epstein-Barr virus (EBV) is a ubiquitous human gamma-herpesvirus that establishes lifelong latency in infected cells (1–4). During latency, the viral genome persists as a chromatinized episome that is subject to host epigenetic regulation (5–7). Tight repression of viral lytic gene expression is essential for immune evasion and long-term persistence, whereas disruption of this control promotes lytic reactivation and contributes to viral dissemination and disease (1). Central to this process is transcriptional regulation of the EBV immediate-early (IE) genes *ZTA* and *RTA*, which function as master regulators that initiate the lytic transcriptional cascade required for viral DNA replication, capsid assembly, and the release of progenies (8–12).

The repressive chromatin state of the *ZTA* and *RTA* promoters, namely ZTAp and RTAp, plays a critical role in controlling EBV latency. In latently infected cells, these promoters are maintained in a transcriptionally restrictive state characterized by low RNA polymerase II occupancy and limited enrichment of activating histone modifications (13, 14). Among these, trimethylation of histone H3 lysine 4 (H3K4Me3) is a key determinant of promoter activation (15). H3K4Me3 is broadly associated with transcriptionally active genes and facilitates recruitment of the transcriptional machinery. During EBV lytic reactivation, rapid accumulation of H3K4Me3 at the *ZTA* and *RTA* promoters accompanies transcriptional activation, whereas removal of this mark promotes latency (14).

Dynamic regulation of H3K4 methylation is controlled by opposing activities of histone methyltransferases and lysine demethylases (16). A recent study showed that the histone demethylase LSD1 (also known as KDM1A) acts as a critical restriction factor for EBV lytic replication by removing the activating H3K4Me3 mark to maintain repressive viral chromatin (17).

RNA-binding proteins have increasingly been recognized as important regulators of viral infection beyond their canonical roles in RNA processing. Heterogeneous nuclear ribonucleoproteins are multifunctional factors that coordinate transcription, RNA metabolism, and chromatin organization. Among them, HNRNPA2B1 has been implicated in the life cycles of multiple viruses, where it can influence viral RNA stability, nuclear export, or host immune response to viral infection (18–26). HNRNPA2B1 has also been linked to transcriptional regulation in host cells, where it acts at the interface of RNA regulation and epigenetic control of chromatin through lncRNA, *HOTAIR* (27, 28). Despite these emerging roles, whether HNRNPA2B1 contributes directly to the repression of viral chromatin in latently infected cells has not been defined.

Recent advances in Clustered Regularly Interspaced Short Palindromic Repeats (CRISPR)-based chromatin targeting have enabled locus-specific identification of proteins associated with defined genomic regions. This approach employs catalytically inactive Cas9 (dCas9) fused to an epitope tag, such as Flag or HA, in a strategy termed engineered DNA-binding molecule-mediated chromatin immunoprecipitation (enChIP). Targeting dCas9 to a specific genomic locus allows affinity purification of the associated chromatin and interacting proteins, which can subsequently be identified by mass spectrometry (MS) (29–32). Applying enChIP-MS to viral episome provides a powerful strategy to uncover host factors that directly regulate viral chromatin. From our enChIP-MS analysis, we discovered that a group of heterogeneous nuclear ribonucleoproteins (HNRNPs) was significantly enriched at EBV ZTAp. In this study, we focused on HNRNPA2B1 for further analysis of its role in EBV latency and reactivation. Our study revealed that HNRNPA2B1 represses EBV IE promoters by recruitment of the histone demethylase LSD1, thus limiting H3K4Me3 accumulation, promoting latency, and restricting lytic reactivation. These results establish a previously unrecognized role for HNRNPA2B1 in viral epigenetic regulation and highlight a mechanism linking RNA-binding proteins to chromatin-based control of EBV latency and reactivation.

## RESULTS

### Identification of HNRNPs as EBV promoter binding proteins using enChIP-MS

Previously, we developed a CRISPR-mediated EBV reactivation (CMER) system using a dCas9-based strategy in which dCas9 was fused to the transcriptional activator VP64 and directed to EBV ZTAp to induce EBV lytic reactivation (33). To extend the utility of this platform, we cloned a non-targeting control sgRNA and a ZTAp-targeting sgRNA (sg5) into the CRISPR/dCas9-Flag vector for enChIP analysis of ZTAp using Akata (EBV+) Burkitt lymphoma cells.

**Fig 1:**
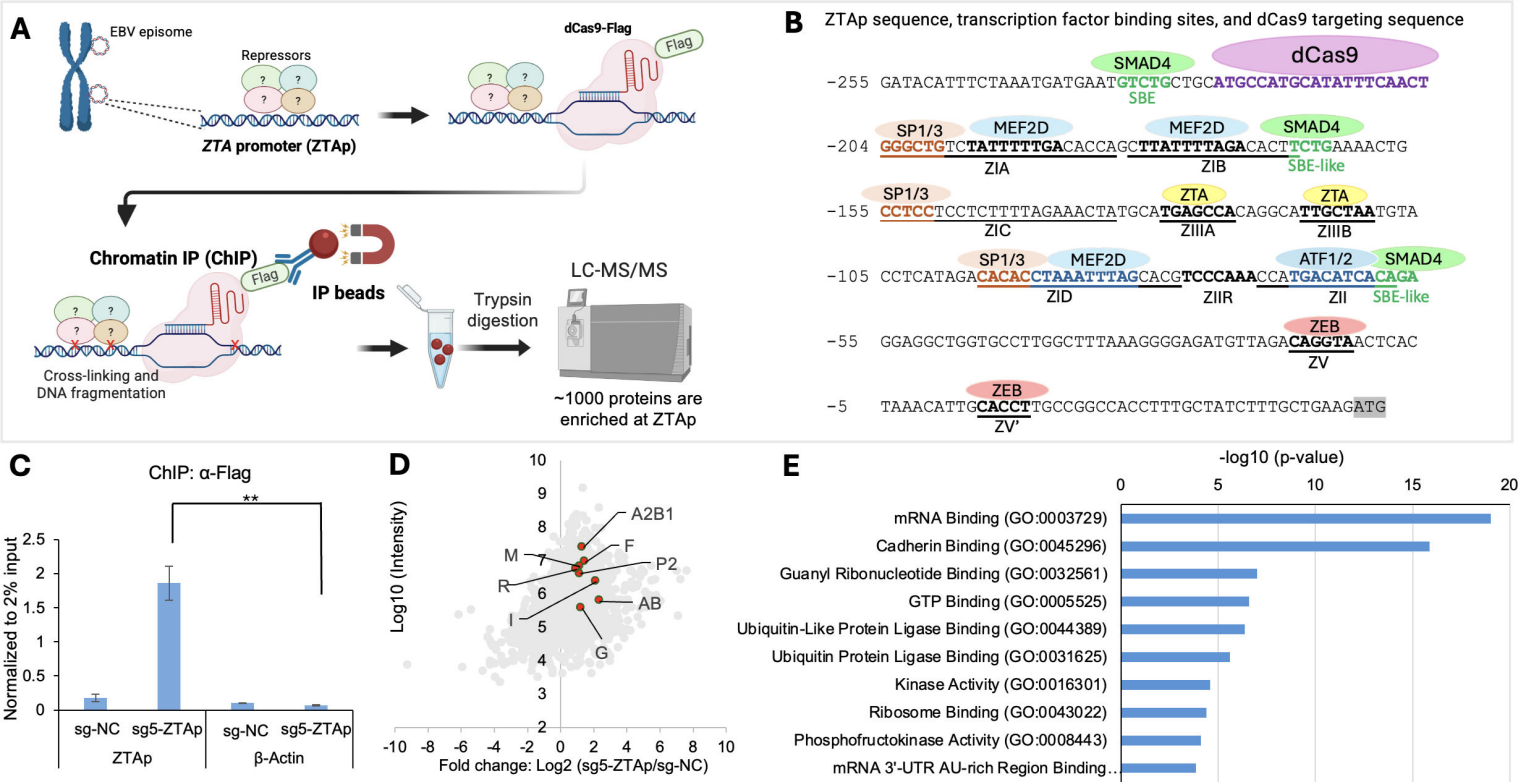
Identification of HNRNP proteins as EBV ZTAp-associated proteins using enChIP-MS. (**A**) Schematic illustration of the experimental workflow of enChIP-MS. Flag-tagged dCas9 is guided to the ZTAp on the EBV episome using a ZTAp-specific sgRNA. Chromatin is crosslinked and fragmented, followed by Flag-based ChIP as described in Materials and Methods. ChIPed proteins are digested with trypsin and analyzed by LC-MS/MS to identify proteins enriched at the ZTAp. (**B**) Schematic representation of ZTAp sequence showing transcription factor binding sites and the dCas9-targeting sequence, as indicated. ZIA, ZIB, ZIC, ZIIIA, ZIIIB, ZID, ZIIR, ZII, ZV, and ZV′ denote cis-acting regulatory elements within the promoter region. SBE denotes SMAD-binding element. (**C**) Akata (EBV+) B cells were used to create cell lines using lentivirus carrying dCas9-Flag with control (sg-NC) and ZTAp-targeting sgRNA (sg5-ZTAp). The enrichment of Flag-dCas9 at the ZTAp and a control β-actin locus was quantified by ChIP-qPCR and normalized to 2% input as described in Materials and Methods. Data are shown as mean ± SD. **P < 0.01. (**D**) Scatter plot showing fold change (log_2_ sg5-ZTAp/sg-NC) versus signal intensity (log_10_) of proteins identified by enChIP-MS. Eight HNRNPs with greater than twofold enrichment at the ZTAp were highlighted. (**E**) Gene ontology analysis of differentially upregulated genes highlighting the top 10 enriched molecular functions.

By employing enChIP-MS, we sought to identify proteins enriched at the ZTAp that may function as transcriptional repressors. In this system, Flag-tagged dCas9 is directed to the ZTAp of the EBV episome, enabling selective enrichment and identification of chromatin-associated proteins at this regulatory region. Following crosslinking, chromatin fragmentation, and Flag-based ChIP, the ZTAp-associated proteins will be subjected to trypsin digestion and then analyzed by liquid chromatography-tandem mass spectrometry (LC-MS/MS) (Fig. 1A).

According to ZTAp sequence, the dCas9-sg5 target site is positioned between SMAD-binding element (SBE) and the ZIA region, relative to previously reported transcription factor binding sites, including SMAD4, SP1/3, MEF2D, ZTA, and ZEB1/2 (8, 34–36) (Fig. 1B).

ChIP followed by quantitative polymerase chain reaction (ChIP-qPCR) analysis confirmed efficient and specific targeting of dCas9 to the ZTAp, with significantly greater enrichment observed in cells expressing the ZTAp-targeting sgRNA (sg5-ZTAp) compared with the non-targeting control sgRNA (sg-NC), while no enrichment was detected at the *β-actin* locus (Fig. 1C). Proteomic analysis identified approximately 1,000 proteins enriched at the ZTAp using twofold enrichment cut-off relative to sg-NC (Table S1). Among these, several proteins previously reported to associate with ZTAp were detected, such as SMAD4 (36), IFI16 (37), and HDAC2 (38). Interestingly, eight HNRNPs were significantly enriched at the ZTAp, suggesting a potential role for RNA-binding proteins in regulating EBV lytic gene expression (Fig. 1D). Consistent with this, gene ontology analysis of ZTAp-enriched proteins revealed a significant overrepresentation of mRNA-binding proteins (Fig. 1E; Table S1).

**Fig 2:**
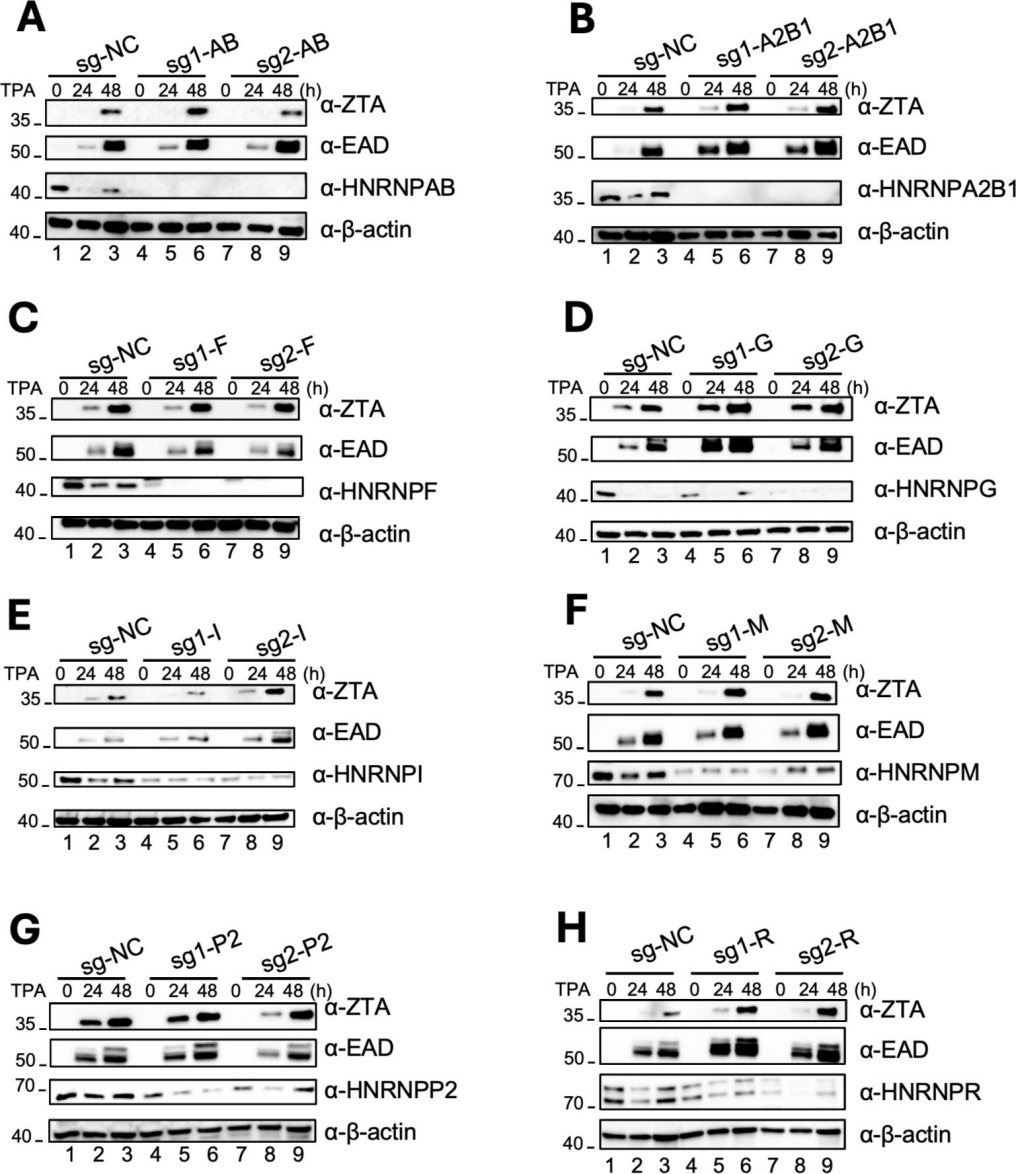
Identification of HNRNP proteins as repressors of EBV lytic reactivation using targeted CRISPR/Cas9-based gene knockout screening. WB analysis of EBV lytic proteins (ZTA and EAD) and HNRNPs in EBV+ SNU-719 cells following depletion of HNRNPAB (**A**), HNRNPA2B1 (**B**), HNRNPF (**C**), HNRNPG (**D**), HNRNPI (**E**), HNRNPM (**F**), HNRNPP2 (**G**), HNRNPR (**H**), and then lytically induced by TPA treatment for 0, 24, and 48 h. β-actin serves as a loading control.

To functionally evaluate the role of these HNRNPs in EBV lytic reactivation, individual HNRNPs were depleted using CRISPR/Cas9-mediated gene targeting in EBV+ gastric carcinoma cells SNU-719 (Fig. 2) (39), an epithelial cell line amenable for CRISPR/Cas9 screening of multiple candidate genes with higher efficiency than B cells. Western blot (WB) analysis revealed that depletion of HNRNPA2B1 (Fig. 2B), HNRNPG (Fig. 2D), and HNRNPR (Fig. 2H) resulted in significantly increased expression of ZTA and the early lytic protein EAD compared to the non-targeting control cells following lytic induction by 12-O-tetradecanoylphorbol-13-acetate (TPA) treatment, whereas depletion of other HNRNPs (AB/F/I/M/P2) did not affect lytic induction (Fig. 2A, C, E through G). These results suggest that multiple HNRNP proteins function as restriction factors that limit EBV lytic gene expression.

### HNRNPA2B1 functions as a conserved repressor for gamma-herpesvirus lytic reactivation

We focused on HNRNPA2B1 for further functional analysis as its role in the EBV life cycle has not been investigated. To evaluate the function of HNRNPA2B1 in EBV lytic reactivation across different cell types, we depleted HNRNPA2B1 in EBV+ B lymphoma cells and nasopharyngeal carcinoma cells.

**Fig 3:**
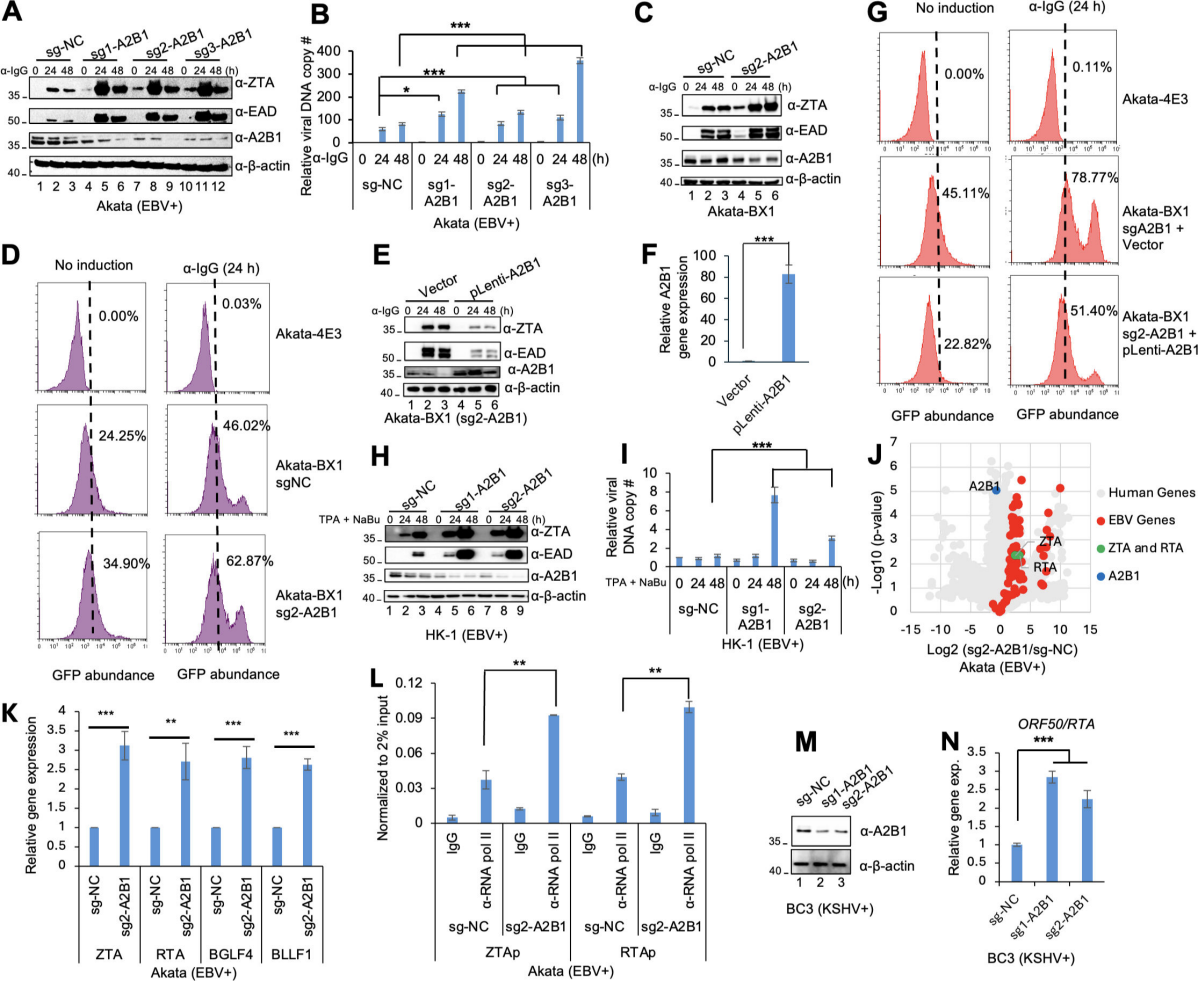
HNRNPA2B1 functions as a conserved repressor for gamma-herpesvirus lytic reactivation. (**A**) WB analysis of EBV lytic proteins ZTA and EAD in Akata (EBV+) cells expressing control sgRNA (sg-NC) or HNRNPA2B1-targeting sgRNAs following lytic induction by IgG-crosslinking for 0, 24, and 48 h. β-actin serves as a loading control. (**B**) Relative extracellular EBV DNA copy number in Akata (EBV+) cells treated as in panel A was quantified by qPCR as described in Materials and Methods. Data are presented relative to sg-NC at 0 h. Data represent ± SD from three biological replicates. *P < 0.05; ***P < 0.001. (**C**) WB analysis of ZTA, EAD, and HNRNPA2B1 expression in Akata-BX1 cells expressing control sgRNA (sg-NC) or HNRNPA2B1-targeting sgRNAs following lytic induction by IgG-crosslinking. (**D**) Flow cytometric analysis of GFP reporter expression in Akata-BX1 cells carrying control sg-NC or HNRNPA2B1-targeting sgRNA (sg2-A2B1) without or with IgG-crosslinking for 24 h. EBV- Akata-4E3 cells were included as a GFP− control. (**E**) WB analysis of EBV lytic protein and HNRNPA2B1 expression in HNRNPA2B1-depleted Akata-BX1 cells (sg2-A2B1) transduced with lentiviruses carrying empty vector or HNRNPA2B1-expressing plasmid (pLenti-A2B1) following lytic induction by IgG-crosslinking. (**F**) RT-qPCR quantification of HNRNPA2B1 transcript levels in Akata-BX1 (sg2-A2B1) cells carrying empty vector or HNRNPA2B1-expressing plasmid established in panel E. Data represent ± SD from three biological replicates. ***P < 0.001. (**G**) Flow cytometric analysis of GFP reporter expression in Akata-BX1 (sg2-A2B1) cells expressing vector control or HNRNPA2B1 without or with IgG-crosslinking for 24 h. EBV-Akata-4E3 cells were included as a GFP− control. (**H**) WB analysis of ZTA and EAD expression in HK-1 (EBV+) cells carrying control sg-NC or HNRNPA2B1-targeting sgRNAs following lytic induction by TPA and sodium butyrate (NaBu) treatment for 0, 24, and 48 h. (**I**) Relative extracellular EBV DNA copy number in HK-1 (EBV+) cells treated as in panel H was quantified by qPCR as described in Materials and Methods. Data are presented relative to sg-NC at 0 h. Data represent ± SD from three biological replicates. ***P < 0.001. (**J**) Volcano plot of RNA-seq data showing differential viral gene expression in uninduced Akata (EBV+) cells expressing control sg-NC or HNRNPA2B1-targeting sgRNA (sg2-A2B1). EBV genes are shown in red; IE genes *ZTA* and *RTA* are shown in green; and HNRNPA2B1 (A2B1) is shown in blue. (**K**) RT-qPCR analysis of EBV lytic gene expression in Akata (EBV+) cells expressing control sg-NC or HNRNPA2B1-targeting sgRNA (sg2-A2B1). Transcripts analyzed include *ZTA*, *RTA*, *BGLF4*, and *BLLF1*. Data are normalized to β-actin and presented as mean ± SD from three biological replicates. **P < 0.01; ***P < 0.001. (**L**) ChIP-qPCR analysis showing enrichment of RNA pol II at ZTAp and RTAp in Akata (EBV+) cells expressing control sg-NC or HNRNPA2B1-targeting sgRNA (sg2-A2B1). Data represent ± SD from three biological replicates. **P < 0.01. (**M**) WB analysis of HNRNPA2B1 expression in BC3 (KSHV+) cells expressing control sg-NC or HNRNPA2B1-targeting sgRNAs as indicated. β-actin serves as a loading control. (**N**) Relative KSHV *ORF50 (RTA*) transcript levels in BC3 (KSHV+) cells expressing control sg-NC or HNRNPA2B1-targeting sgRNAs were quantified by RT-qPCR as described in Materials and Methods. Data are normalized to β-actin and presented as mean ± SD from three biological replicates. The value of sg-NC was set as 1. ***P < 0.01.

In Akata (EBV+) Burkitt lymphoma cells, loss of HNRNPA2B1 led to a significant increase in expression of the IE protein ZTA and the early lytic protein EAD following lytic induction by IgG-crosslinking of B-cell receptor (BCR), compared with cells expressing a control sgRNA (Fig. 3A, lanes 5, 6, 8, 9, 11, and 12 vs lanes 2 and 3). Consistent with WB analysis, quantification of extracellular EBV genome copy number further confirmed a significant increase following HNRNPA2B1 depletion (Fig. 3B). Consistent results were also observed in EBV+ Akata-BX1 cells, where loss of HNRNPA2B1 led to enhanced ZTA and EAD protein expression following BCR stimulation (Fig. 3C, lanes 5 and 6 vs lanes 2 and 3). Akata-BX1 cells express a GFP marker under a CMV promoter, enabling quantification of the population undergoing viral DNA replication by flow cytometry (40). We found a substantial increase in the proportion of GFP+ Akata-BX1 cells upon HNRNPA2B1 depletion, indicating elevated EBV DNA replication at the individual cell level with or without lytic induction (Fig. 3D) with Akata-4E3, an isogenic EBV- Akata cell line, as a control without GFP signal (41). Conversely, overexpression of HNRNPA2B1 in HNRNPA2B1-depleted Akata-BX1 cells suppressed IgG-crosslinking-induced ZTA and EAD protein accumulation (Fig. 3E and F). This repression was accompanied by a marked decrease in GFP+ cells, further supporting that HNRNPA2B1 restricts EBV lytic replication (Fig. 3G).

In HK-1 (EBV+) nasopharyngeal carcinoma cells (42, 43), depletion of HNRNPA2B1 enhanced ZTA and EAD protein expression following lytic induction by TPA and sodium butyrate treatment (Fig. 3H, lanes 5, 6, 8, and 9 vs lanes 2 and 3), and significantly increased the extracellular EBV genome copy number (Fig. 3I).

To further define the role of HNRNPA2B1 in the transcriptional regulation of EBV lytic genes, we performed global gene expression analysis in uninduced Akata (EBV+) cells carrying control sg-NC and HNRNPA2B1-targeting sgRNA (sg2-A2B1). Our RNA sequencing (RNA-seq) analysis revealed widespread upregulation of EBV lytic genes upon HNRNPA2B1 depletion, with IE genes such as *ZTA* and *RTA* among the induced transcripts (Fig. 3J).

To validate the transcriptomic data, we performed RT-qPCR analysis and found that the expression of IE (ZTA/RTA), early (BGLF4), and late (BLLF1) genes is significantly increased in Akata (EBV+) cells depleted of HNRNPA2B1 compared with control cells (Fig. 3K). We reasoned that this is due to enhanced transcriptional activity of viral genes. To test this hypothesis, we assessed RNA polymerase II (RNA Pol II) binding to EBV IE gene promoters, ZTAp and RTAp. We showed significant enrichment of RNA Pol II binding on ZTAp and RTAp relative to IgG control when HNRNPA2B1 is depleted, indicating that loss of HNRNPA2B1 promotes the transcription of EBV lytic genes (Fig. 3L).

To determine whether HNRNPA2B1 plays a role in the reactivation of another gamma-herpesvirus, Kaposi’s sarcoma-associated herpesvirus (KSHV), we depleted HNRNPA2B1 in KSHV+ BC3 primary effusion lymphoma cells (44). WB analysis confirmed effective depletion of HNRNPA2B1 in BC3 (KSHV+) cells (Fig. 3M, lanes 2 and 3 vs lane 1). Correspondingly, RT-qPCR analysis demonstrated increased expression of the KSHV IE gene *ORF50* (*RTA*) following HNRNPA2B1 depletion (Fig. 3N). These results indicate that HNRNPA2B1 broadly suppresses the transcription of herpesvirus lytic genes.

### HNRNPA2B1 binds to EBV and KSHV IE promoters

**Fig 4:**
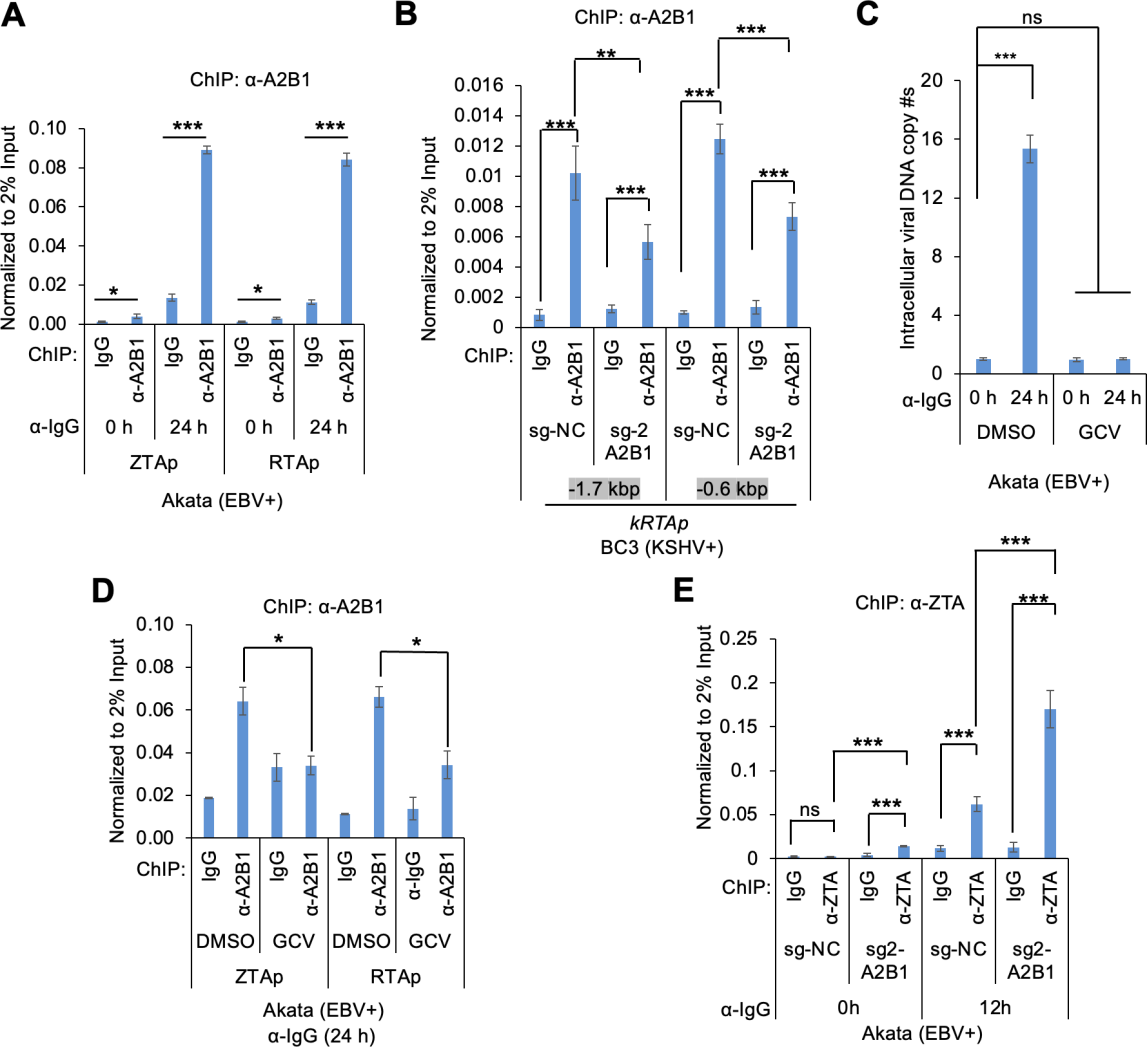
HNRNPA2B1 binds to EBV and KSHV IE gene promoters. (**A**) ChIP-qPCR analysis of HNRNPA2B1 occupancy at ZTAp and RTAp in Akata (EBV+) cells following lytic induction by IgG-crosslinking for 0 and 24 h. Anti-HNRNPA2B1 antibody was used for HNRNPA2B1 ChIP, and nonspecific IgG was used as a negative control. Data are presented as mean ± SD from three biological replicates. *P < 0.05; **P < 0.01. (**B**) ChIP-qPCR analysis of HNRNPA2B1 occupancy at the KSHV RTAp (kRTAp) at two distinct regions (−1.7 and −0.6 kbp relative to transcription start site) in BC3 (KSHV+) cells. An anti-HNRNPA2B1 antibody was used for ChIP, with nonspecific IgG as a negative control. Data are presented as mean ± SD from three biological replicates. **P < 0.01; ***P < 0.001. (**C**) Quantification of intracellular viral DNA copy number in Akata (EBV+) cells under dimethyl sulfoxide (DMSO) solvent control or GCV pretreatment followed by lytic induction for 0 and 24 h. Viral DNA levels were measured by qPCR as described in Materials and Methods. Data are presented as mean ± SD from three biological replicates. **P < 0.01; ns, not significant. (**D**) ChIP-qPCR analysis of HNRNPA2B1 enrichment at ZTAp and RTAp in lytically induced Akata (EBV+) cells pretreated with control DMSO or GCV. Anti-HNRNPA2B1 antibody was used for HNRNPA2B1 ChIP, and nonspecific IgG was used as a negative control. Data are presented as mean ± SD from three biological replicates. *P < 0.05. (**E**) ChIP-qPCR analysis of ZTA enrichment at ZTAp in Akata (EBV+) cells comparing control and HNRNPA2B1-depleted conditions in the absence (0 h) or presence of lytic induction (12 h) by IgG-crosslinking. An anti-ZTA antibody was used for ZTA ChIP, with nonspecific IgG as a negative control. Data are shown as mean ± SD from three biological replicates. ns, not significant; ***P < 0.001.

Our enChIP-MS analysis suggested that HNRNPA2B1 binds to ZTAp. HNRNPA2B1 has previously been reported to bind the herpes simplex virus 1 (HSV-1) genome during primary infection (18). We hypothesize that HNRNPA2B1 restricts EBV lytic replication by binding to IE gene promoters, analogous to the mechanism we previously described for PIAS1 (45). To test this hypothesis, we performed HNRNPA2B1 ChIP followed by qPCR analysis in Akata (EBV+) cells. We observed significant enrichment of HNRNPA2B1 at EBV IE promoters (ZTAp and RTAp) compared with IgG controls, with binding markedly increased at 24 h following lytic induction by IgG cross-linking (Fig. 4A). We also detected HNRNPA2B1 occupancy at two distinct regions of the KSHV RTA promoter (kRTAp) in BC3 (KSHV+) cells (Fig. 4B). The increased binding of HNRNPA2B1 following IgG cross-linking in Akata (EBV+) cells led us to hypothesize that newly synthesized viral DNA may contribute to this enhanced association. To test this possibility, we conducted ChIP analysis in ganciclovir (GCV)-treated lytically induced Akata (EBV+) cells (Fig. 4C and D). As expected, GCV treatment effectively repressed EBV DNA replication (Fig. 4C) (33, 46), and this repression coincided with the reduction of HNRNPA2B1 binding to the ZTAp and RTAp (Fig. 4D). Together, these results suggested that HNRNPA2B1 binds to EBV IE gene promoters in latency and during lytic reactivation.

As a consequence of reduced HNRNPA2B1 occupancy at ZTAp and increased ZTA expression in HNRNPA2B1-depleted cells, we hypothesized that ZTA binding at ZTAp would be further enhanced under HNRNPA2B1 knockdown conditions. To test this, we performed ZTA ChIP at ZTAp at 0 and 12 h post-IgG cross-linking. We observed increased ZTA enrichment at ZTAp in HNRNPA2B1-depleted cells at both time points (Fig. 4E).

### HNRNPA2B1 restricts EBV lytic reactivation by modulating H3K4 trimethylation

We demonstrated that HNRNPA2B1 binds to EBV IE gene promoters, and that HNRNPA2B1 depletion enhances RNA polymerase II occupancy at these promoters and thereby promotes the transcription of EBV IE genes. These observations prompted us to investigate whether loss of HNRNPA2B1 also affects epigenetic marks associated with transcriptional activation.

**Fig 5:**
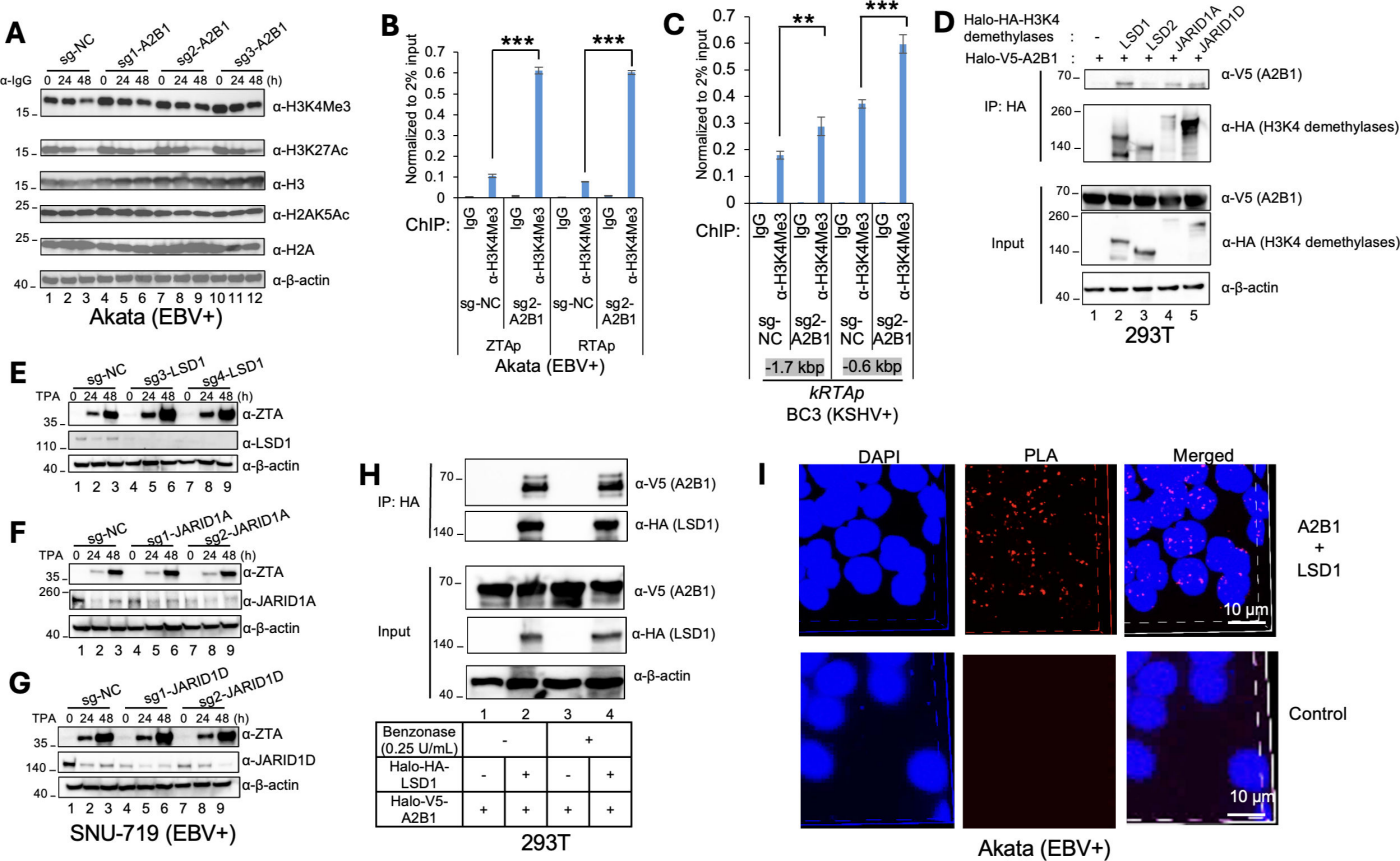
HNRNPA2B1 restricts EBV lytic reactivation by modulating H3K4Me3. (**A**) WB analysis of histone modifications in Akata (EBV+) cells following HNRNPA2B1 depletion and IgG-crosslinking-induced lytic reactivation. Whole-cell lysates were collected at the indicated time points and probed for H3K4Me3, H3K27Ac, total H3, H2AK5Ac, and total H2A. β-actin serves as a loading control. (**B**) ChIP-qPCR analysis of H3K4Me3 enrichment at EBV ZTAp and RTAp in control (sg-NC) and HNRNPA2B1-depleted (sg2-A2B1) Akata (EBV+) cells. Data are shown as relative to 2% of input. Data are presented as mean ± SD from three biological replicates. ***P < 0.001. (**C**) ChIP-qPCR analysis of H3K4Me3 enrichment at KSHV RTAp (kRTAp) at two distinct regions in control (sg-NC) and HNRNPA2B1-depleted (sg2-A2B1) BC3 (KSHV+) cells. Data are shown as relative to 2% of input. Data are presented as mean ± SD from three biological replicates. **P < 0.01; ***P < 0.001. (**D**) Plasmids expressing Halo-HA-H3K4 demethylases and Halo-V5-HNRNPA2B1 were co-transfected into HEK-293T cells as indicated. Cell lysates containing the indicated tagged proteins were IP-ed with anti-HA antibody-conjugated beads and analyzed by WB with anti-V5 and anti-HA antibodies. Co-IP analysis showing stronger interaction between HNRNPA2B1 and LSD1. β-actin serves as a loading control. (**E–G**) WB analysis of EBV lytic protein ZTA and LSD1 (**E**), JARID1A (**F**), or JARID1D (**G**) expression in LSD1-, JARID1A-, or JARID1D-depleted SNU-719 cells following lytic induction by TPA treatment. β-actin serves as a loading control. (**H**) Co-IP analysis validating the interaction between HNRNPA2B1 and LSD1. HEK-293T cells were transfected with Halo-HA-LSD1 and Halo-V5-HNRNPA2B1 as indicated. Cell lysates were treated with or without benzonase, followed by IP using anti-HA antibody-conjugated beads. IP and input samples were analyzed by WB with anti-V5 and anti-HA antibodies. β-actin serves as a loading control. (**I**) Proximity ligation assay (PLA) demonstrating the interaction between HNRNPA2B1 and LSD1 *in situ*. Akata (EBV+) cells were blocked with 3% bovine serum albumin (BSA) in phosphate-buffered saline (PBS) for 1 h at room temperature. Subsequently, the cells were incubated with either PBS control or a combination of mouse anti-LSD1 and rabbit anti-HNRNPA2B1 antibodies. Probes were then added for ligation and amplification. Cell nuclei were visualized using Nikon AXR after staining with 4′,6-diamidino-2-phenylindole (DAPI). The interaction between HNRNPA2B1 and LSD1 *in situ* was indicated by red dots representing PLA signals. Scale bars, 10 µm.

Interestingly, we found that HNRNPA2B1 depletion globally increased the activating histone mark H3K4Me3, with comparatively smaller effects on other transcription-associated marks, including H3K27ac and H2AK5ac (Fig. 5A, lanes 4–6, 7–9, 10–12 vs lanes 1–3). To determine whether HNRNPA2B1 directly influences H3K4Me3 level at EBV IE gene promoters, we performed ChIP qPCR analysis of H3K4Me3 occupancy at EBV ZTAp and RTAp. We found that loss of HNRNPA2B1 significantly enhanced H3K4Me3 enrichment at both IE promoters in uninduced Akata (EBV+) cells (Fig. 5B). Similarly, we observed enhanced H3K4Me3 enrichment at kRTAp when HNRNPA2B1 is depleted in BC3 (KSHV+) cells (Fig. 5C), indicating that HNRNPA2B1 normally suppresses this activating histone mark to limit gamma-herpesviruses IE gene expression.

### HNRNPA2B1 interacts with H3K4 demethylases

Because H3K4Me3 is dynamically regulated by methyltransferases and demethylases, we reasoned that HNRNPA2B1 interacts with H3K4 demethylases to limit H3K4Me3 levels. To identify which H3K4 demethylases associate with HNRNPA2B1, we cloned multiple demethylases involved in H3K4 demethylation and assessed their interactions with HNRNPA2B1 by co-immunoprecipitation (co-IP). These experiments revealed that HNRNPA2B1 interacts with LSD1, LSD2, and JARID1D, with the strongest binding observed for LSD1 (Fig. 5D, lane 2 vs lanes 4 and 5).

To evaluate whether these H3K4 demethylases play a role in EBV reactivation, we performed CRISPR Cas9-mediated depletion of LSD1, JARID1A, or JARID1D individually in SNU-719 cells (Fig. 5E through G). We found that depletion of LSD1 resulted in a marked increase in ZTA expression following lytic induction (Fig. 5E), whereas knockdown of JARID1A or JARID1D had minimal effects (Fig. 5F and G). These results are consistent with a prior report identifying LSD1 as a key restriction factor for EBV lytic replication (17). Therefore, we focused on the interaction between HNRNPA2B1 and LSD1 in EBV reactivation.

To determine whether the interaction between HNRNPA2B1 and LSD1 is mediated by nucleic acids, cell lysates were treated with benzonase prior to co-IP. We observed that benzonase treatment did not affect the association between HNRNPA2B1 and LSD1, indicating that their interaction is independent of nucleic acids (Fig. 5H, lane 4 vs lane 2).

To demonstrate the interaction between HNRNPA2B1 and LSD1 in a physiologically relevant context, we performed proximity ligation assays (PLAs) in Akata (EBV+) cells. Strong PLA signals localized predominantly to the nucleus were detected (Fig. 5I), supporting the physiological relevance of the HNRNPA2B1-LSD1 interaction.

**Fig 6:**
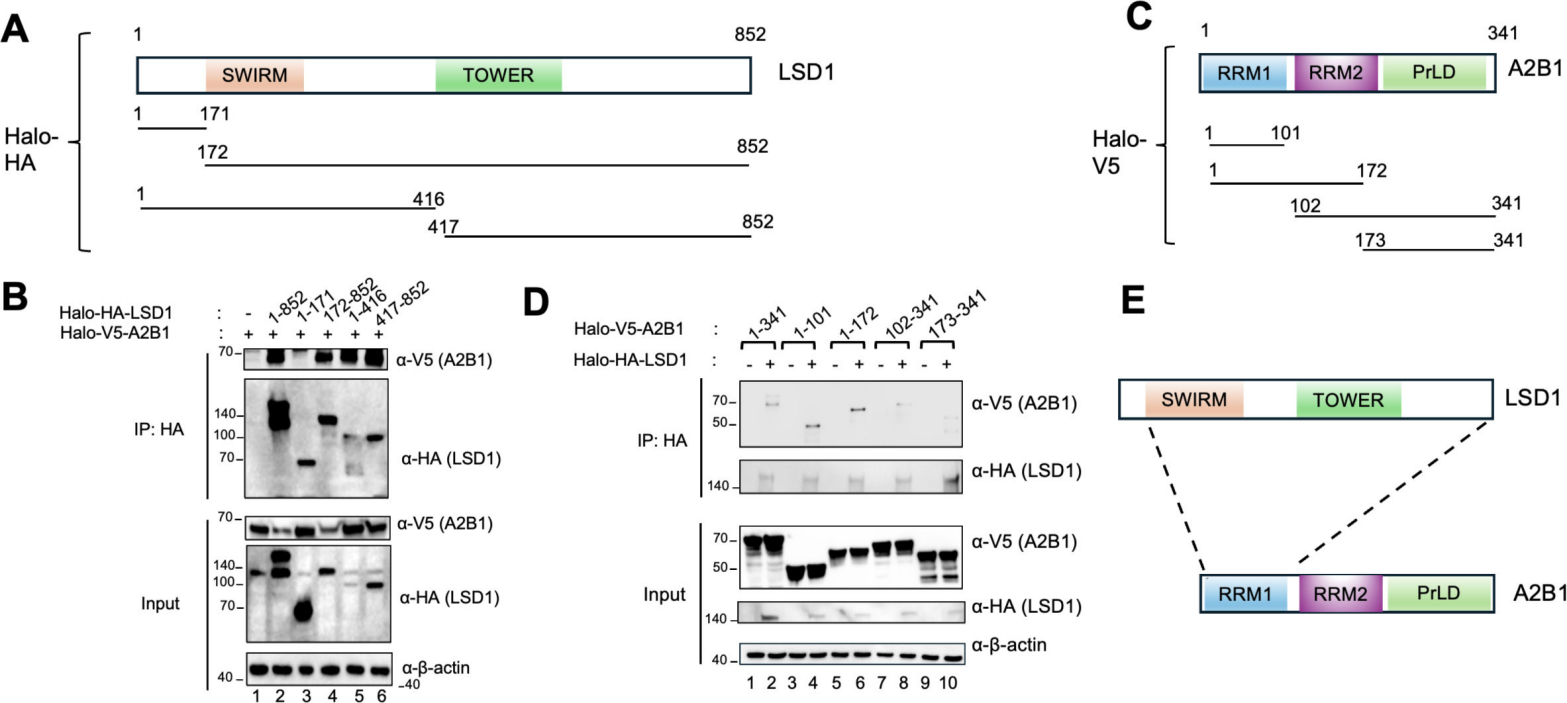
Domain-specific interaction between LSD1 and HNRNPA2B1. (**A**) Schematic representation of LSD1 domain architecture and Halo-HA-tagged LSD1 truncation constructs. SWIRM: Swi3p, Rsc8p, and Moira domain; TOWER: a pair of long, antiparallel α-helices containing domain. (**B**) HEK-293T cells were co-transfected with Halo-V5-HNRNPA2B1 and either full-length or truncated LSD1 constructs as indicated. Cell lysates containing the indicated tagged proteins were IP-ed with anti-HA antibody-conjugated beads and analyzed by WB with anti-V5 and anti-HA antibodies. β-actin serves as a loading control. (**C**) Schematic representation of HNRNPA2B1 domain organization and Halo-V5-tagged HNRNPA2B1 truncation constructs. PrLD, prion-like domain; RRM, RNA recognition motif. (**D**) HEK-293T cells were co-transfected with full-length Halo-HA-LSD1 and either full-length or truncated Halo-V5-HNRNPA2B1 constructs as indicated. Cell lysates containing the indicated tagged proteins were IP-ed with anti-HA antibody-conjugated beads and analyzed by WB with anti-V5 and anti-HA antibodies. β-actin serves as a loading control. (**E**) Proposed model illustrating the domains required for the interaction between LSD1 and HNRNPA2B1.

To define the regions of LSD1 required for its interaction with HNRNPA2B1, we performed co-IP assays in HEK-293T cells transfected with plasmids expressing full-length Halo-V5-HNRNPA2B1 together with either full-length Halo-HA-LSD1 or individual Halo-HA-tagged fragments (Fig. 6A). Full-length LSD1 and all LSD1 fragments except the N-terminal region (amino acids 1–171) co-IPed with V5-HNRNPA2B1 (Fig. 6B, lanes 2, 4, 5, and 6). These results indicate that domains covering both SWIRM and TOWER regions of LSD1 mediate the interaction with HNRNPA2B1.

To determine the regions of HNRNPA2B1 involved in LSD1 binding, HEK-293T cells were co-transfected with plasmids expressing full-length Halo-HA-LSD1 and either full-length Halo-V5-HNRNPA2B1 or individual Halo-V5-tagged fragments (Fig. 6C). Immunoprecipitation of LSD1 using anti-HA antibody-conjugated beads demonstrated that the N-terminal regions of HNRNPA2B1 (amino acids [aa] 1–101 or 1–172) were strongly co-IPed with HA-LSD1, whereas the C-terminal regions (aa 102–341 or 173–341) showed much weaker association (Fig. 6D, lanes 4 and 6 vs lanes 8 and 10). Together, these data suggest that the C-terminal domain of LSD1 interacts specifically with the RNA recognition motif 1 (RRM1) of HNRNPA2B1 (Fig. 6E).

### HNRNPA2B1 recruits LSD1 to EBV IE promoters

**Fig 7:**
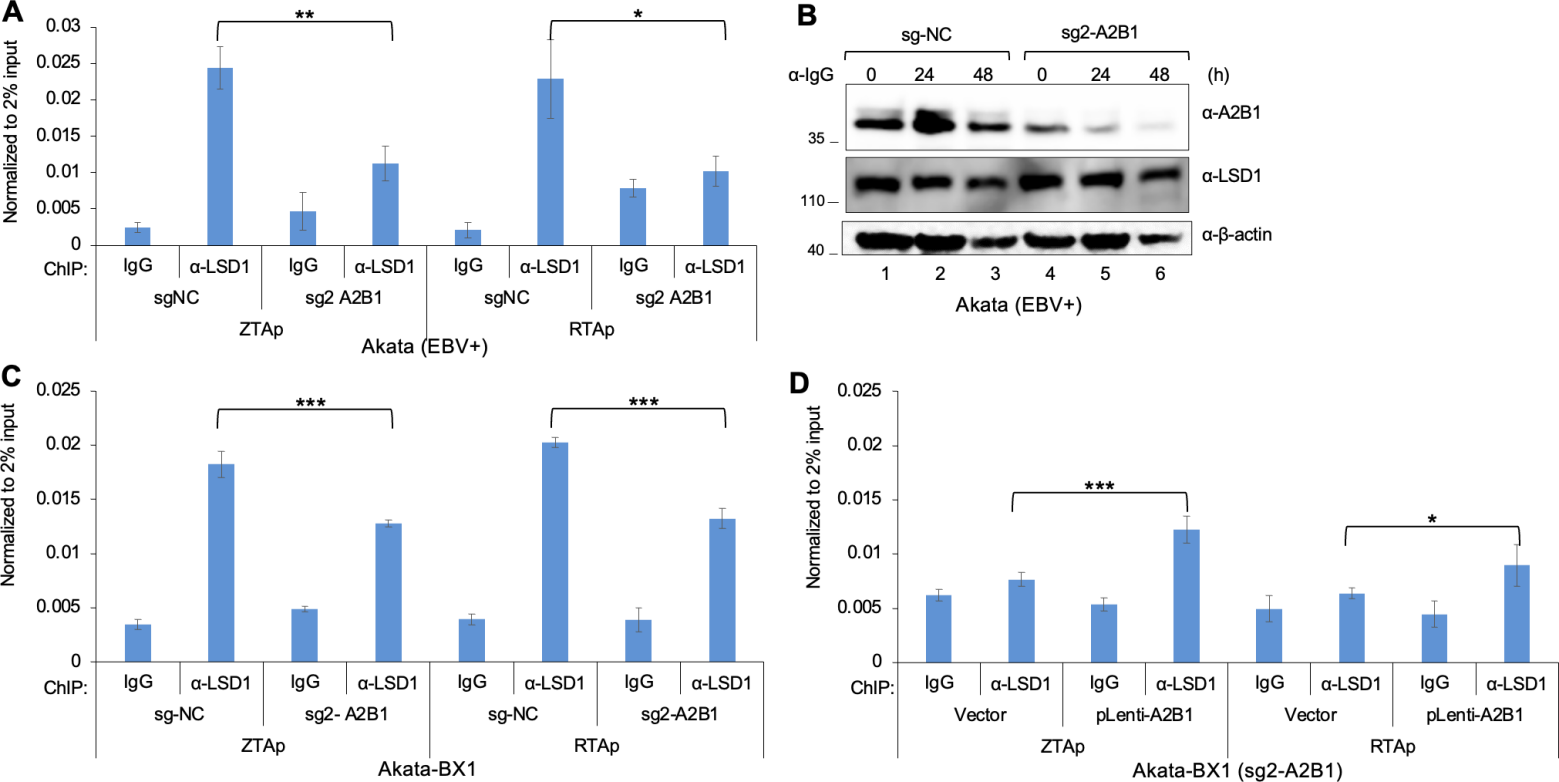
HNRNPA2B1 promotes LSD1 recruitment to EBV IE gene promoters. (**A**) ChIP-qPCR analysis of LSD1 occupancy at ZTAp and RTAp in uninduced Akata (EBV+) cells transduced with non-targeting control (sg-NC) or HNRNPA2B1-targeting sgRNA (sg2-A2B1). Anti-LSD1 was used for LSD1 ChIP and IgG served as a negative control. Data are normalized to 2% input and shown as mean ± SD from three biological replicates. *P < 0.05; **P < 0.01. (**B**) Akata (EBV+) cells carrying control sg-NC or HNRNPA2B1-targeting sgRNA (sg2-A2B1) were treated with anti-human IgG for the indicated times. Protein levels of HNRNPA2B1 and LSD1 were analyzed by WB. β-actin serves as a loading control. (**C**) ChIP-qPCR analysis of LSD1 occupancy at ZTAp and RTAp in uninduced Akata-BX1 cells expressing control sg-NC or HNRNPA2B1-targeting sgRNA (sg2-A2B1). Anti-LSD1 was used for LSD1 ChIP and IgG served as a negative control. Data are normalized to 2% input and shown as mean ± SD from three biological replicates. ***P < 0.001. (**D**) ChIP-qPCR analysis of LSD1 occupancy at ZTAp and RTAp in uninduced Akata-BX1 sgA2B1 cells reconstituted with vector control or HNRNPA2B1 (pLenti-A2B1). Anti-LSD1 antibody was used for LSD1 ChIP and IgG served as a negative control. Data are normalized to 2% input and shown as mean ± SD from three biological replicates. *P < 0.05; ***P < 0.001.

We hypothesize that HNRNPA2B1 recruits LSD1 to EBV IE gene promoters to limit H3K4Me3 levels. To test this hypothesis, we performed ChIP-qPCR analysis of LSD1 enrichment at ZTAp and RTAp in control and HNRNPA2B1-depleted uninduced Akata (EBV+) cells. We found that loss of HNRNPA2B1 resulted in a significant reduction of LSD1 occupancy at ZTAp and RTAp (Fig. 7A). Because LSD1 expression was not affected by HNRNPA2B1 depletion (Fig. 7B, lanes 4–6 vs lanes 1–3), the reduced LSD1 promoter occupancy in HNRNPA2B1-depleted cells reflects impaired recruitment rather than LSD1 abundance.

Consistent with the findings in Akata (EBV+) cells, depletion of HNRNPA2B1 in uninduced Akata-BX1 cells led to reduced LSD1 binding at both ZTAp and RTAp (Fig. 7C). To further confirm these results, we restored HNRNPA2B1 expression in HNRNPA2B1-depleted uninduced Akata-BX1 cells. We noticed that HNRNPA2B1 restoration resulted in enhanced recruitment of LSD1 to ZTAp and RTAp, as determined by LSD1 ChIP-qPCR analysis (Fig. 7D). Together, these results demonstrate that HNRNPA2B1 fosters the recruitment of LSD1 to EBV ZTAp and RTAp, thereby contributing to the maintenance of a repressive chromatin state to maintain viral latency and restrict lytic reactivation.

### HNRNPA2B1 interacts with CoREST complex

We demonstrated that HNRNPA2B1 physically associates with LSD1 and facilitates its recruitment to EBV IE gene promoters. Given that LSD1 commonly functions within the Co-repressor of Repressor Element-1 Silencing Transcription (CoREST) complex through interactions with canonical partners such as ZNF217 or RCOR1 (17), we hypothesized that HNRNPA2B1 may be associated with these components either directly or within the same chromatin-associated complex. To evaluate this possibility, we performed PLA to assess HNRNPA2B1-ZNF217 and HNRNPA2B1-RCOR1 interactions *in situ*.

**Fig 8:**
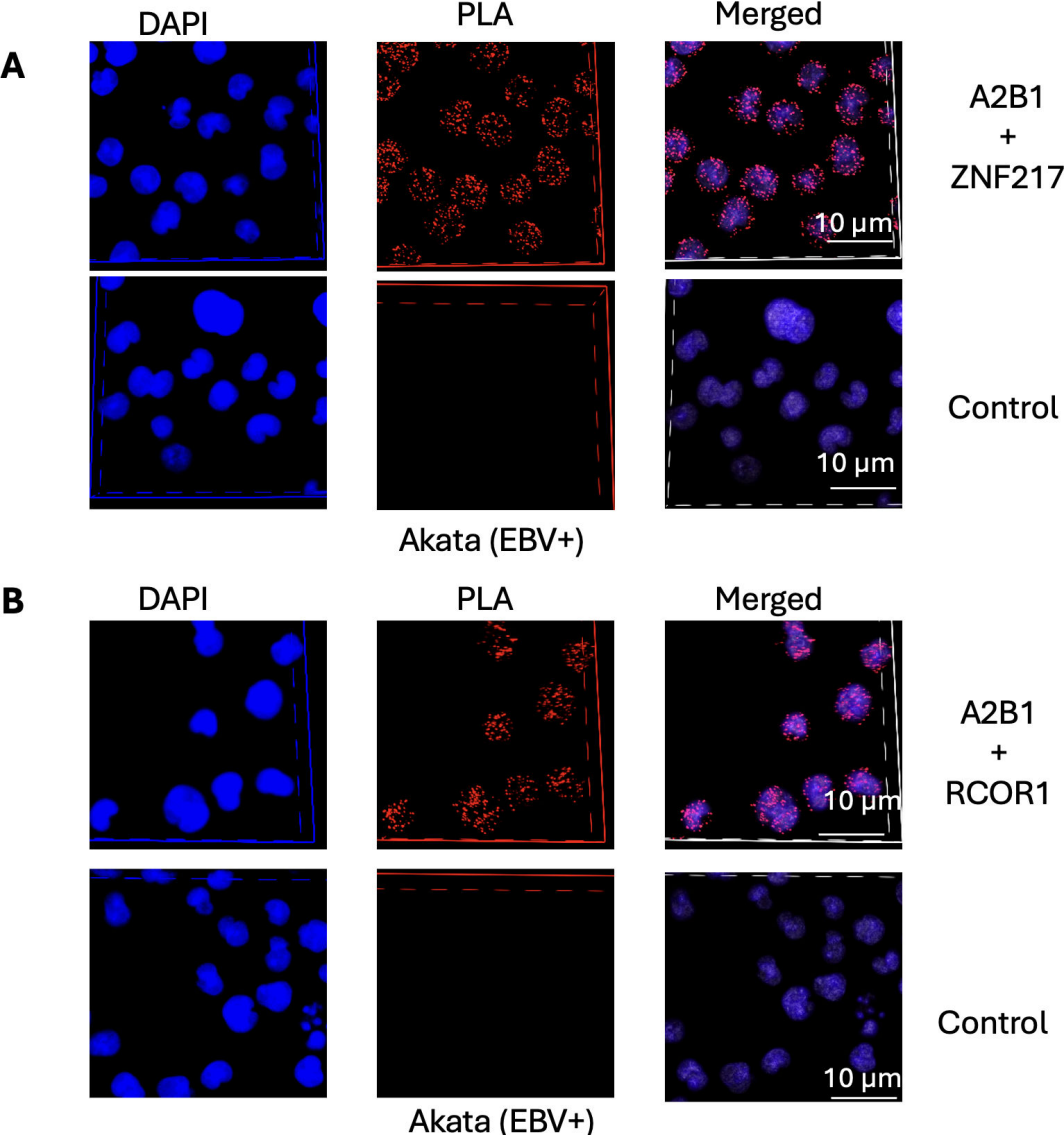
HNRNPA2B1 interacts with ZNF217 and RCOR1. PLA demonstrating the interaction between HNRNPA2B1 and ZNF217 (**A**) or RCOR1 (**B**) in Akata (EBV+). Interactions between HNRNPA2B1 and ZNF217, as well as HNRNPA2B1 and RCOR1, are indicated by punctate red PLA signals, reflecting protein proximity at the subcellular level. Scale bars, 10 μm.

PLA analysis revealed strong and predominantly nuclear signals for both HNRNPA2B1-ZNF217 (Fig. 8A) and HNRNPA2B1-RCOR1 (Fig. 8B) interactions. The nuclear enrichment of these signals is consistent with the known localization and function of the CoREST complex in chromatin-mediated transcriptional repression. These findings support the model that HNRNPA2B1 may function in concert with, or as part of, the LSD1-containing CoREST complex, potentially contributing to the repression of IE gene transcription through chromatin remodeling.

**Fig 9:**
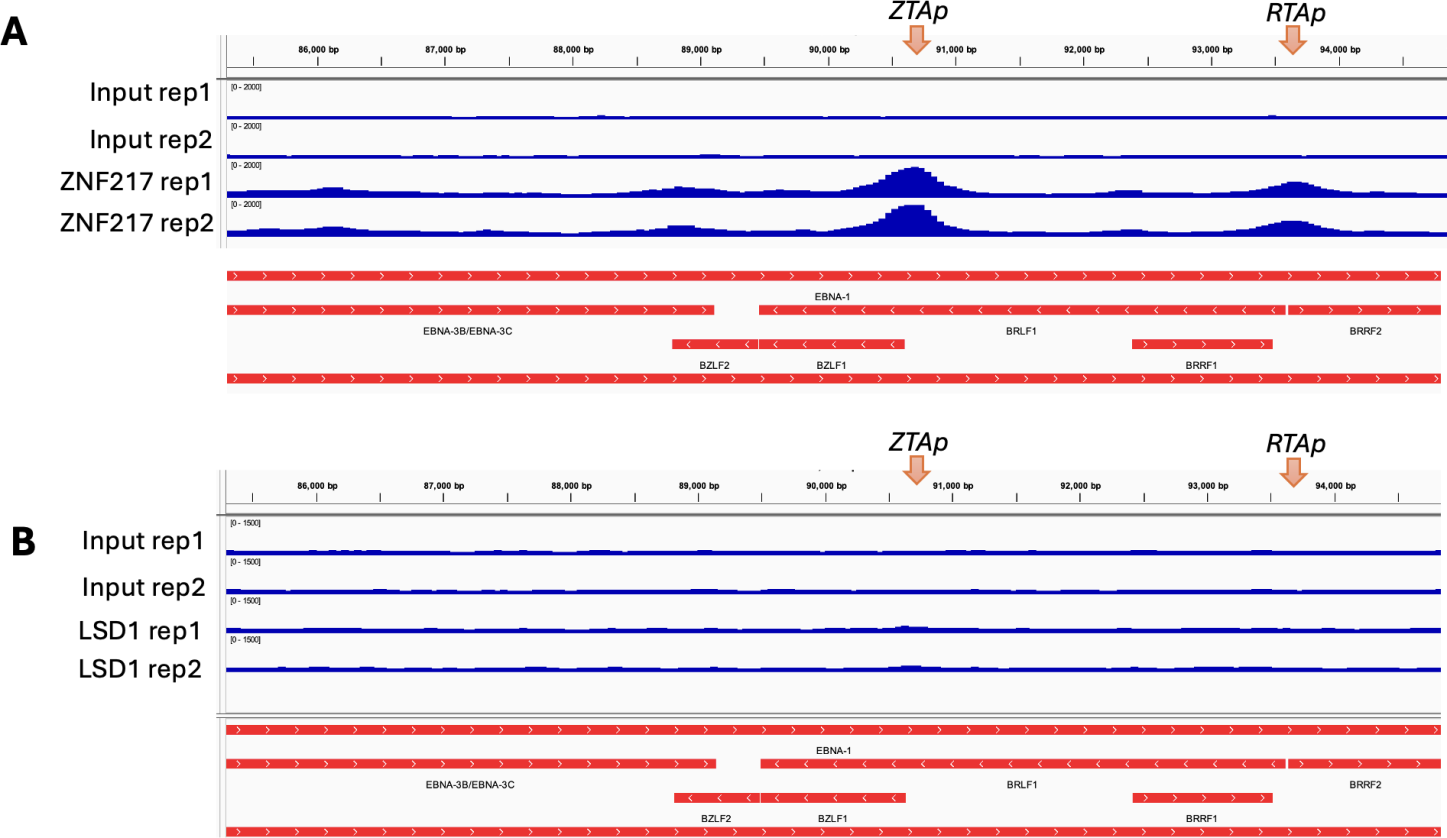
ZNF217 is highly enriched at EBV ZTAp and RTAp. Genome browser views showing ChIP-seq signal tracks across the EBV genomic region encompassing *ZTA* (*BZLF1*) and *RTA* (*BRLF1*) loci. The upper panel displays robust enrichment of ZNF217 (**A**), with prominent peaks centered at the ZTAp and RTAp regions. In contrast, the lower panel shows comparatively weak and diffuse binding of LSD1 (**B**) across the same loci. Signal intensity is represented on the *y*-axis, and genomic coordinates (bp) are shown along the *x*-axis.

Given that HNRNPA2B1 is not known to bind double-stranded DNA (dsDNA) in a sequence-specific manner (47, 48), its association with ZTAp and RTAp may occur indirectly, potentially through interaction with ZNF217. To further explore this relationship, we re-analyzed previously generated ChIP-seq data from Liao et al. (17) and observed strong enrichment of ZNF217 at both ZTAp and RTAp. In contrast, LSD1 displayed comparatively weaker occupancy at these loci (Fig. 9A vs B).

These observations suggest that ZNF217 may serve as a primary DNA-binding factor that facilitates recruitment of HNRNPA2B1 to EBV promoters, while LSD1 may be present as part of a secondary or more dynamic complex. This model is consistent with the limited intrinsic dsDNA-binding affinity of HNRNPA2B1 and supports a cooperative mechanism in which protein-protein interactions and chromatin context contribute to its localization at target gene promoters.

## DISCUSSION

EBV latency is maintained by stringent repression of IE gene expression, yet the host factors that promote this silencing at the viral chromatin level remain incompletely defined (5–7). Using enChIP-MS, we directly captured host proteins associated with EBV ZTAp in its native chromatin context, facilitating unbiased identification of putative factors involved in *ZTA* gene repression. This approach has previously been used to identify the nucleosome remodeling and deacetylase complex at D4Z4 repeat in myoblasts, host proteins associated with the parvovirus B19 genome, and EPAS1 promoter-associated proteins in neuroblastoma cells, supporting its utility for locus-specific chromatin proteomics (30–32). Among approximately 1,000 proteins identified, we focused on HNRNPs for further functional analysis and discovered a previously unappreciated role for HNRNPA2B1 in EBV latency and reactivation.

HNRNPA2B1 has been reported as an anti-viral or pro-viral factor in different contexts. For example, HNRNPA2B1 restricts hepatitis B virus (HBV) and SARS-CoV-2 replication by triggering anti-viral immune response via TBK1-IRF3 pathway (21). During HSV-1 infection, HNRNPA2B1 promotes interferon production via binding to viral DNA and promoting the trafficking of *CGAS*, *IFI16*, and *STING* mRNAs, thereby restricting viral replication (18). In contrast, overexpression of HNRNPA2B1 in THP-1 cells has been shown to enhance gene expression for several viruses, including severe fever with thrombocytopenia syndrome virus (SFTSV) (26). In the Senecavirus A model, the interaction between viral VP3 protein and HNRNPA2B1 facilitates viral internal ribosome entry site (IRES)-driven protein translation while simultaneously suppressing host cell translation and interferon response (22).

In our study, we demonstrated that HNRNPA2B1 consistently suppresses EBV lytic reactivation across multiple EBV+ cell models. Intriguingly, we found that depletion of HNRNPA2B1 enhances expression of IE and downstream lytic genes, increases RNA polymerase II occupancy at EBV IE promoters, increases the frequency of cells entering the lytic cycle, and promotes the production of extracellular viral genomes. These effects were observed in EBV+ cancer models derived from both B cells and epithelial cells, indicating that HNRNPA2B1-mediated viral gene repression is not cell type specific. Conversely, ectopic expression of HNRNPA2B1 suppressed lytic replication, supporting a direct role for HNRNPA2B1 in maintaining viral latency.

One novel aspect from our study is the establishment of HNRNPA2B1 as a regulator of viral chromatin accessibility. Loss of HNRNPA2B1 led to increased enrichment of activating histone marks, particularly H3K4Me3, at the IE promoters, consistent with enhanced transcriptional activity. We further showed that HNRNPA2B1 interacts with the histone demethylase LSD1 in a nucleic acid-independent manner and is required for efficient recruitment of LSD1 to EBV IE promoters. Disruption of this pathway reduced LSD1 occupancy, promoted accumulation of H3K4Me3 mark, and facilitated lytic transcription.

LSD1, a core component of the CoREST complex, functions canonically as a histone demethylase that regulates H3K4 methylation. Recent work has shown that recruitment of LSD1 to the ZTA promoter depends on the transcription factor ZNF217 to regulate H3K4 methylation level (17). Our PLAs revealed spatial proximity between HNRNPA2B1 and ZNF217/RCOR1, key components of the CoREST complex. Given that ZNF217 has been previously implicated in binding to the ZTA promoter and restricting EBV reactivation, these findings raise the possibility that HNRNPA2B1 may function in coordination with the CoREST complex to modulate chromatin modifications, including H3K4Me3, at lytic gene promoters. However, additional biochemical and functional studies will be required to define the precise molecular architecture and regulatory hierarchy of this complex.

HNRNPA2B1 plays a pivotal role in a variety of cellular processes, particularly RNA metabolism (49, 50). While HNRNPA2B1 is primarily recognized for its RNA-binding capabilities, our findings, together with recent studies, suggest that HNRNPA2B1 may also function as a chromatin-associated factor to control gene expression (27, 28, 51). Our findings expand this functional repertoire by demonstrating a direct role of HNRNPA2B1 in chromatin-based transcriptional repression of a latent viral genome. The observation that HNRNPA2B1 associates with IE promoters and regulates RNA polymerase II recruitment suggests a coordinated control of transcription initiation and early RNA processing by HNRNPA2B1.

The transcriptional changes induced by HNRNPA2B1 depletion indicate that its role in EBV reactivation is possibly not limited to direct regulation of viral promoters. Instead, loss of HNRNPA2B1 could result in broad remodeling of host gene expression programs. Although our RNA-seq analysis did not identify a dominant set of differentially expressed anti-viral restriction factors or chromatin regulators, it remains possible that more subtle or combinatorial changes across multiple pathways collectively influence the cellular environment required for efficient lytic reactivation. Such distributed effects may include modulation of RNA processing, protein translation, or stress-associated signaling pathways that are not easily captured by single-gene enrichment analysis. Therefore, HNRNPA2B1 possibly regulates EBV latency and reactivation through an integrated mechanism involving both direct chromatin-associated functions at viral promoters and indirect effects mediated by host gene network reprogramming. Notably, we did not observe dysregulation of type I interferon expression in our system, in contrast to prior reports (18).

A limitation of the present study is that the observed increase in H3K4Me3 enrichment at EBV IE promoters following HNRNPA2B1 depletion may reflect both direct and indirect mechanisms. In particular, elevated H3K4Me3 levels at ZTAp and RTAp could arise as a downstream consequence of increased expression of these lytic transactivators, which themselves promote chromatin activation during viral reactivation. Therefore, the current experimental framework does not fully distinguish whether HNRNPA2B1 directly regulates histone modification at these promoters or whether the observed chromatin changes are secondary to lytic gene expression. Addressing this question would require systems with genetically blocked lytic transactivation (e.g., *ZTA*- or *RTA*-deficient EBV) to distinguish the primary epigenetic role of HNRNPA2B1 from indirect effects of reactivation.

**Fig 10:**
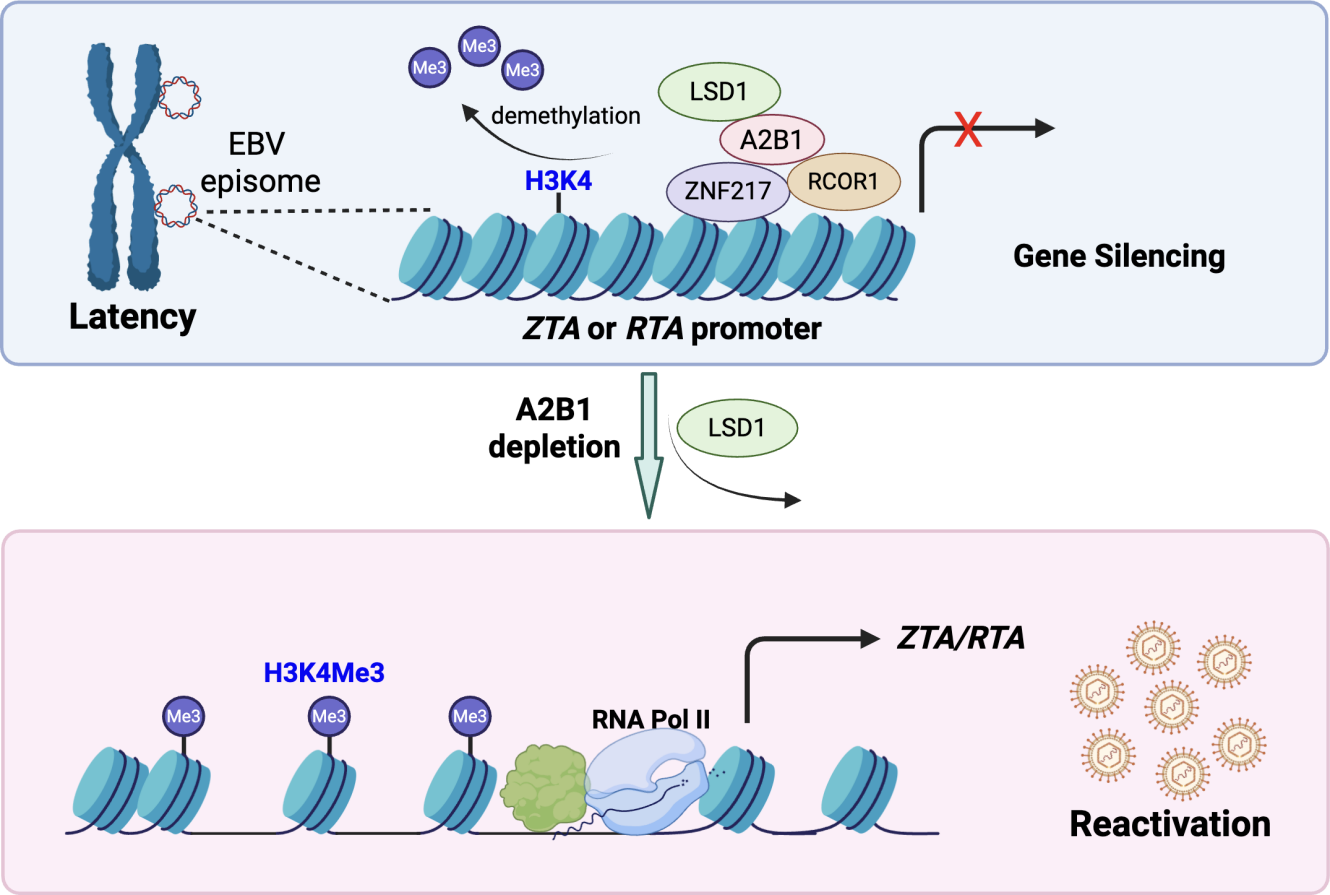
Model summarizing the role of HNRNPA2B1 in promoting EBV latency and suppressing lytic reactivation. HNRNPA2B1 (A2B1), in coordination with ZNF217 and RCOR1, recruits LSD1 to EBV *ZTA* and *RTA* promoters, leading to H3K4 demethylation and repression of lytic gene expression, thereby maintaining viral latency. Depletion of HNRNPA2B1 diminishes LSD1 recruitment to EBV IE gene promoters, increases H3K4Me3 levels, and enhances RNA polymerase II occupancy, resulting in activation of EBV lytic gene expression and viral reactivation.

In summary, this study identifies HNRNPA2B1 as a previously unrecognized epigenetic regulator that coordinates with the CoREST complex to restrict EBV lytic reactivation through LSD1-dependent histone H3K4 demethylation at IE promoters (Fig. 10). These findings provide new insights into how host RNA-binding proteins interact with a histone demethylase complex to control herpesvirus gene expression and highlight a latency control mechanism that may be broadly relevant to other latent DNA viruses.

## MATERIALS AND METHODS

### Cell lines and cultures

Akata (EBV+), SNU-719 (EBV+), HK-1 (EBV+), Akata-BX1 (EBV+), and BC3 (KSHV+) cells were cultured in Roswell Park Memorial Institute medium (RPMI 1640) supplemented with 10% FBS (Cat. #26140079, Thermo Fisher Scientific, Waltham, MA, USA) in 5% CO_2_ at 37°C (45, 52–55). HK-1 (EBV+) cells (courtesy of Dr. George Tsao, Hong Kong University) were supplemented with 800 μg/mL G418 in the culture medium (33, 42, 43, 56). Akata-BX1 (EBV+) cells were supplemented with 495 μg/mL G418 in the culture medium (40). HEK-293T cells were cultured in Dulbecco’s modified Eagle medium (DMEM) supplemented with 10% FBS in 5% CO_2_ at 37°C (57, 58). See also Table 1 for cell line sources.

**TABLE 1:** Antibody, reagents, constructs, and cell lines

Reagent or resource	Source	Identifier
Antibodies and reagents		
Mouse anti-HNRNPA2B1	Proteintech	Cat. #67445-1-Ig
Rabbit anti-HNRNPA2B1	Proteintech	Cat. #14813-1-AP
Rabbit anti-HNRNPAB	Proteintech	Cat. #31175-1-AP
Rabbit anti-HNRNPF	Proteintech	Cat. #14974-1-AP
Rabbit anti-HNRNPG	Cell Signaling Technology	Cat. #14794T
Rabbit anti-HNRNPI	Proteintech	Cat. #12582-1-AP
Rabbit anti-HNRNPM	Proteintech	Cat. #26897-1-AP
Rabbit anti-HNRNPP2	Proteintech	Cat. #11570-1-AP
Rabbit anti-HNRNPR	Proteintech	Cat. #29980-1-AP
Mouse anti-ZTA (BZ1)	Santa Cruz	Cat. #sc-53904
Mouse anti-RNA polymerase II	Millipore Sigma	Cat. #05-623-25UG
Mouse IgG	Santa Cruz	Cat. #sc-2025
Rabbit anti-EBV EA-D-p52/50 antibody, clone R3	Millipore Sigma	Cat. #MAB8186
Mouse anti-β-actin antibody	Santa Cruz	Cat. #sc-47778
Anti-V5-HRP	Thermo Fisher	Cat. #R960-25
Anti-HA	Roche	Cat. #11-867-431-001
Rabbit anti-H3K4Me3	Cell Signaling Technology	Cat. #9751S
Acetyl-Histone Antibody Sampler Kit	Cell Signaling Technology	Cat. #9933
JARID1/KDM5 Histone Demethylase Antibody Sampler Kit	Cell Signaling Technology	Cat. #25497T
Rabbit anti-H3K27Ac	Cell Signaling Technology	Cat. #4353S
Rabbit anti-H3 (D2B12)	Cell Signaling Technology	Cat. #4620S
Rabbit anti-KDM1A (LSD1)	Cell Signaling Technology	Cat. #2139S
Rabbit anti-RCOR1	Proteintech	Cat. #27686-1-AP
Mouse anti-ZNF217	Proteintech	Cat. #67730-1-Ig
Mouse anti-KDM1A (LSD1)	Proteintech	Cat. #67037-1-Ig
ChromoTek DYKDDDDK Fab-Trap Agarose beads	Proteintech	Cat. #ffa
Goat anti-human IgG (whole molecule)	MP Biomedicals	Cat. #0855087
Phorbol-12-myristate-13-acetate	Millipore Sigma	Cat. #52-440-01MG
Sodium butyrate	TCI America	Cat. #S051925G
PEI Max	Polyscience	Cat. #24765-100
Anti-V5 magnetic beads	MBL	Cat. #M167-11
Anti-HA magnetic beads	Thermo Fisher	Cat. #88836
SimpleChIP Enzymatic Chromatin IP Kit (Magnetic Beads)	Cell Signaling Technology	Cat. #9003S
RQ1 RNase-free DNase	Promega	Cat. #M6101
Proteinase K	Meridian Bioscience	Cat. #BIO-37084
ISOLATE II RNA Mini Kit	Meridian Bioscience	Cat. #BIO-52073
High-Capacity cDNA Reverse Transcription Kit with RNase Inhibitor	Applied Biosystems	Cat. #4374966
NaveniFlex Cell Red Kit	Navinci	Cat. #NC.MR.100
Ganciclovir	Millipore Sigma	Cat. #PHR1593
Constructs		
pMD2.G	Addgene	Plasmid #12259
psPAX2	Addgene	Plasmid #12260
pHTN-V5-HNRNPA2B1	This study	pFS80
pLenti-HNRNPA2B1	This study	pFS112
pHTN-HA-KDM1A (LSD1)	This study	pFS574
pHTN-HA-KDM1B (LSD2)	This study	pFS688
pHTN-HA-KDM5A (JARID1A)	This study	pFS689
pHTN-HA-KDM5D (JARID1D)	This study	pFS687
Cell lines		
Akata (EBV+)	Hayward Lab Collection	Not applicable
SNU-719	Hayward Lab Collection	Not applicable
Akata-BX1	Hayward Lab Collection	Not applicable
293T cells	Hayward Lab Collection	Not applicable
BC3	Hayward Lab Collection	Not applicable
HK-1 (EBV+)	(42, 43, 56)	Not applicable

### Plasmid construction

The non-targeting control (sg-NC) and ZTAp-targeting sg5 oligonucleotides from our previous study (33) were cloned into the pZLCv2-3xFLAG-dCas9-HA-2xNLS vector (Cat. #106357, Addgene; gift from Stephen Tapscott) using methods as previously described (30). HNRNPA2B1, LSD1, JARID1A, JARID1D, and LSD2 coding sequences were amplified by PCR using Q5 High-Fidelity polymerase from the following templates: MBP-hnRNPA2_FL_WT (Cat. #98662, Addgene; gift from Nicolas Fawzi), pIDS-LSD1fl (Cat. #109157, Addgene; gift from Monika Golas), TFORF2771-KDM5A (JARID1A) (Cat #143614, Addgene; gift from Feng Zhang), TFORF2768-KDM5D (JARID1D) (Cat. #142159, Addgene; gift from Feng Zhang), and pLenti6.3/V5-DEST-KDM1B (LSD2) (Cat. HsCD00963160, DNAsu). The PCR products were cloned into the pHTN-CMV-Neo vector. HNRNPA2B1 was tagged with V5 at the N-terminus, while all KDM constructs carried an N-terminal HA tag. HNRNPA2B1, LSD1, and LSD2 were constructed using the Gibson assembly method, whereas JARID1A and JARID1D were inserted into the vector following EcoRI/NotI double digestion and DNA ligation. All primer sequences are listed in Table 2.

**TABLE 2:** Primer list

Primer name	Sequence (5′ to 3′)	Note
pFS_31_F	CACCGTCTCTTGCTACAGCACGTTT	sg1 HNRNPA2B1
pFS_31_R	AAACAAACGTGCTGTAGCAAGAGAC	sg1 HNRNPA2B1
pFS_32_F	CACCGACTCTCCCATCAATTGAATG	sg2 HNRNPA2B1
pFS_32_R	AAACCATTCAATTGATGGGAGAGTC	sg2 HNRNPA2B1
pFS_33_F	CACCGCAGTTCCGTAAGCTCTTTAT	sg3 HNRNPA2B1
pFS_33_R	AAACATAAAGAGCTTACGGAACTGC	sg3 HNRNPA2B1
pFS_491_F	caccgCCGTGGAAACCGCAACCG	sg1 HNRNPAB
pFS_491_R	AAACCGGTTGCGGTTTCCACGGC	sg1 HNRNPAB
pFS_492_F	CACCGCCCTCTTGACCGTCCAGTGT	sg2 HNRNPAB
pFS_492_R	AAACACACTGGACGGTCAAGAGGGC	sg2 HNRNPAB
pFS_497_F	CACCGTTCGTGCAGTTTGCCTCGC	sg1 HNRNPF
pFS_497_R	AAACGCGAGGCAAACTGCACGAAC	sg1 HNRNPF
pFS_498_F	CACCGAGTGCCGACAGCGCCAACGA	sg2 HNRNPF
pFS_498_R	AAACTCGTTGGCGCTGTCGGCACTC	sg2 HNRNPF
pFS_507_F	CACCGCTTACCATCTTGACGTGCAT	sg1 HNRNPM
pFS_507_R	AAACATGCACGTCAAGATGGTAAGC	sg1 HNRNPM
pFS_508_F	CACCGTAGACCAATGCACGTCAAGA	sg2 HNRNPM
pFS_508_R	AAACTCTTGACGTGCATTGGTCTAC	sg2 HNRNPM
pFS_509_F	CACCGACCTTGATCTTCGCTTCATC	sg1 HNRNPR
pFS_509_R	AAACGATGAAGCGAAGATCAAGGTC	sg1 HNRNPR
pFS_510_F	CACCGACCTGATGAAGCGAAGATCA	sg2 HNRNPR
pFS_511_R	AAACTGATCTTCGCTTCATCAGGTC	sg2 HNRNPR
pFS_525_F	CACCGCCTTAAGCTCCTGTATCACG	sg1 HNRNPG
pFS_525_R	AAACCGTGATACAGGAGCTTAAGGC	sg1 HNRNPG
pFS_526_F	CACCGCACCTTTCCATTCATGTCTC	sg2 HNRNPG
pFS_526_R	AAACGAGACATGAATGGAAAGGTGC	sg2 HNRNPG
pFS_527_F	CACCGCTTCAACACTGTGCCGAACT	sg1 HNRNPI
pFS_527_R	AAACAGTTCGGCACAGTGTTGAAGC	sg1 HNRNPI
pFS_528_F	CACCGCTCCGTGTTCATCTCGATGA	sg2 HNRNPI
pFS_528_R	AAACTCATCGAGATGAACACGGAGC	sg2 HNRNPI
pFS_529_F	CACCGTATGGCTCGACTGGCGGCTA	sg1 HNRNPP2
pFS_529_R	AAACTAGCCGCCAGTCGAGCCATAC	sg1 HNRNPP2
pFS_530_F	CACCGCGCCAGTCGAGCCATATCCC	sg2 HNRNPP2
pFS_530_R	AAACGGGATATGGCTCGACTGGCGC	sg2 HNRNPP2
pFS_69_F	ATGGGTAAGCCTATCCCTAACCCTCTCCTCGGTCTCGATTCTACG ATGGAACGTGAAAAAGAGCA	Halo-V5-HNRNPA2B1
pFS_69_R	GGCCCAAATCTAGATATCCGTTAGTAACGGCTGCGGCCAC	Halo-V5-HNRNPA2B1
pFS_441_F	ATGTACCCATACGATGTTCCAGATTACGCTATGTTATCTGGGAAGAAGG	Halo-HA-LSD1
pFS_441_R	CAGCAGTCCCCAAGCATGTGAGGATATCTAGATTTGGGCC	Halo-HA-LSD1
pFS_614_F	ATGTACCCATACGATGTTCCAGATTACGCTATGGCTACACCTCGGGGCAGAACTAAG	Halo-HA-LSD2
pFS_614_R	GGCCCAAATCTAGATATCCGTTAAAATGCAGCGATCTTGCTGGC	Halo-HA- LSD2
pFS_605_F	CGCTTCCGAATTCAGAGCTCAACCGATGTACCCATACGATGTTCCAGATTACGCTATGATGGCGGGCGTGGGGCCGGGGGGCTACG	Halo-HA-JARID1A
pFS_605_R	AAGCGGCCGCAAAATCCTGCAGGAATTGGGCCCAAATCTAGATATCCGCTAACTGGTCTCTTTAAGATCCTCCATTG	Halo-HA-JARID1A
pFS_604_F	CGCTTCCGAATTCAGAGCTCAACCGATGTACCCATACGATGTTCCAGATTACGCTATGATGGAACCGGGGTGTGACGAG	Halo-HA-JARID1D
pFS_604_R	AAGCGGCCGCAAAATCCTGCAGGAATTGGGCCCAAATCTAGATATCCGCTACAACTGTTGCTCATCAGAGTAGG	Halo-HA-JARID1D
pFS_486_F	CACCGAGAGCCGACTTCCTCATGAC	sg3 LSD1
pFS_486_R	AAACGTCATGAGGAAGTCGGCTCTC	sg3 LSD1
pFS_632_F	CACCGTTTCTGAAACAGGATCGTGT	sg4 LSD1
pFS_632_R	AAACACACGATCCTGTTTCAGAAAC	sg4 LSD1
pFS_625_F	CACCGATACACCAATGTCGGTCCGG	sg1 JARID1A
pFS_625_R	AAACCCGGACCGACATTGGTGTATC	sg1 JARID1A
pFS_626_F	CACCGTGTCCTAAATGTGTCGCCG	sg2 JARID1A
pFS_626_R	AAACCGGCGACACATTTAGGACAC	sg2 JARID1A
pFS_627_F	CACCGTTGTTTCAAAGACTTACCGC	sg1 JARID1D
pFS_627_R	AAACGCGGTAAGTCTTTGAAACAAC	sg1 JARID1D
pFS_628_F	CACCGAAAGACTTACCGCGGGTGGG	sg2 JARID1D
pFS_628_R	AAACCCCACCCGCGGTAAGTCTTTC	sg2 JARID1D
RL0068	CATCTTCAGCAAAGATAGCAAAGG	ZTAp qPCR
RL0069	GGGCTGTCTATTTTTGACACCAGC	ZTAp qPCR
RL0070	GGATTACTGGTCTTTTATGAGCC	EBV RTAp qPCR
RL0071	TTGACTGCAATATTTCCTCCAG	EBV RTAp qPCR
RL0066	TGGACTTCGAGCAAGAGATG	β-actin qPCR
RL0067	GAAGGAAGGCTGGAAGAGTG	β-actin qPCR
pFS_701_F	GTAACCAGGGTGGTGGTTATG	HNRNPA2B1 qPCR
pFS_701_R	CTCGGTTGCTGGTTGTAGTT	HNRNPA2B1 qPCR
RL0062	AGTCCTTCTTGGCTAGTCTGTTGAC	BALF5 qPCR
RL0063	CTTTGGCGCGGATCCTC	BALF5 qPCR
pFS_814_F	GATCGGCGAAGTGGATAGAGT	KSHV RTAp −1.7 qPCR
pFS_814_R	CCCTATTGGTCACATCTCACG	KSHV RTAp −1.7 qPCR
pFS_815_F	AAGACACTGACCCACCAAGG	KSHV RTAp −0.6 qPCR
pFS_815_R	GGTGCCACCAATGTATGACC	KSHV RTAp −0.6 qPCR

### Generation of stable cell line

Lentiviruses isolated from the HEK293-T medium were used to infect the Akata (EBV+), HK-1 (EBV+), and SNU-719 (EBV+) cells. Forty-eight hours post-transduction, the cells were cultured in the presence of puromycin (2 µg/mL) or blasticidin (10 μg/mL) for cell line establishment.

### enChIP-MS

enChIP-MS was modified from a previously described protocol (30). 4 × 10^7^ cells were collected by centrifugation at 1,000 rpm for 5 min at room temperature using a swinging bucket rotor. The cell pellet was resuspended in 1 mL of ice-cold Cell Lysis Buffer (10 mM Tris-HCl pH 8.0, 10 mM NaCl, 0.2% NP-40, EDTA-free protease inhibitor, and PMSF) and incubated on ice for 10 min to release nuclei.

Nuclei were pelleted at 2,500 rpm for 5 min at 4°C in a swinging bucket centrifuge and resuspended in 37 mL room temperature PBS. Crosslinking was performed by adding 1.5 mL of 37% formaldehyde to achieve a final concentration of 1.5%, followed by gentle rocking for 15 min at room temperature. The reaction was quenched by adding 4 mL of 10× glycine (Cat. #9003S, Cell Signaling Technology) and incubating for an additional 5 min at room temperature with rocking. Crosslinked nuclei were pelleted at 2,000 rpm for 10 min at 4°C, resuspended in 1 mL cold PBS, transferred to a 1.5 mL tube, and centrifuged again at 2,000 rpm for 5 min at 4°C.

The supernatant was removed, and the pellet was washed with 2 mL ice-cold 1× Buffer B (Cat. #9003S, Cell Signaling Technology) supplemented with 1 µL DTT, followed by centrifugation and removal of the supernatant.

The nuclei were then resuspended in 200 µL 1× Buffer B containing 0.1 µL DTT and transferred to a 1.5 mL microcentrifuge tube. Micrococcal nuclease digestion was initiated by adding 5 µL enzyme, mixing gently by inversion, and incubating at 37°C for 20 min with mixing every 3 min. The reaction was stopped by adding 10 µL EDTA and placing the tube on ice for 2 min. Nuclei were pelleted at 16,000 × *g* for 1 min at 4°C, and the supernatant was discarded.

The nuclear pellet was resuspended in 200 µL 1× ChIP Buffer (Cat. #9003S, Cell Signaling Technology) supplemented with 1 µL of 200× protease inhibitor cocktail (Cat. #9003S, Cell Signaling Technology) and incubated on ice for 10 min. Samples were sonicated using 10-s pulse on and 10-s pulse off cycles for five rounds, with 30 s incubation on ice between rounds. Lysates were clarified by centrifugation at 9,400 × *g* for 10 min at 4°C.

For immunoprecipitation, we utilized ChromoTek DYKDDDDK Fab-Trap Agarose beads (Cat. #ffa, Proteintech). Briefly, 160 µL FLAG-Trap beads was equilibrated in 2 mL IP Wash I buffer (10 mM Tris-HCl pH 7.5, 150 mM NaCl, and 0.25% NP-40) and centrifuged at 2,500 × *g* for 2 min at 4°C. The wash was repeated once.

The clarified chromatin lysate was diluted with 300 µL 1× ChIP Buffer supplemented with 1.5 µL 200× protease inhibitor cocktail and incubated overnight at 4°C with the prepared beads. Beads were collected by centrifugation at 2,500 × *g* for 2 min at 4°C and washed twice with 2 mL ice-cold IP Wash I buffer.

Beads were subsequently washed three times with 2 mL ice-cold IP Wash II buffer (10 mM Tris-HCl pH 7.5 and 150 mM NaCl), each followed by centrifugation at 2,500 × *g* for 2 min at 4°C. After the final wash, beads were resuspended in 160 µL ice-cold IP Wash II buffer, divided equally into two tubes of approximately 80 µL each, centrifuged again at 2,500 × *g* for 2 min at 4°C, and the supernatant was removed. Samples were stored at −80°C for downstream MS analysis by the Taplin Mass Spectrometry Facility at Harvard Medical School.

Beads were washed at least five times with 100 µL of 50 mM ammonium bicarbonate. Modified sequencing-grade trypsin (Promega, Madison, WI, USA) was added at 5 µL (200 ng/µL), and samples were incubated at 37°C overnight for proteolytic digestion. Following digestion, samples were centrifuged or placed on a magnetic rack, depending on bead type, and the supernatant was collected. Peptide extracts were dried using a SpeedVac concentrator for approximately 1 h, resuspended in 50 µL of HPLC solvent A (2.5% acetonitrile and 0.1% formic acid), and desalted using STAGE tips (59).

On the day of analysis, samples were reconstituted in 10 µL of HPLC solvent A. Peptides were separated using a nano-scale reverse-phase HPLC system equipped with a capillary column packed in-house with 2.6 µm C18 spherical silica beads in a fused silica capillary (100 µm inner diameter, approximately 30 cm in length) with a flame-drawn tip (60). After column equilibration, samples were loaded using a Famos autosampler (LC Packings, San Francisco, CA, USA). Peptides were eluted using a linear gradient of increasing concentrations of solvent B (97.5% acetonitrile and 0.1% formic acid).

Eluting peptides were ionized by electrospray ionization and analyzed on a Velos Orbitrap Elite ion trap mass spectrometer (Thermo Fisher Scientific). Peptides were detected, isolated, and fragmented to generate tandem mass spectra. Peptide sequences and corresponding protein identities were assigned by database searching of the acquired spectra using Sequest (Thermo Fisher Scientific) (61). Databases included reversed sequence entries to estimate false discovery rates, and peptide identifications were filtered to achieve a false discovery rate of 1–2% (see Table S1).

### ChIP-qPCR

ChIP process and qPCR were performed as previously described (62). DNA-protein complexes were immunoprecipitated with anti-RNA polymerase II (Cat. #05-623-25UG, Millipore Sigma), anti-HNRNPA2B1 (Cat. #67445-1-Ig, Proteintech), anti-H3K4Me3 (Cat. #9751S, Cell Signaling Technology), anti-KDM1A (LSD1) (Cat. #2139S, Cell Signaling Technology), rabbit IgG control (Cat. #2729, Cell Signaling Technology), anti-ZTA(BZ1) (Cat. #sc-53904, Santa Cruz), and mouse IgG control (Cat. #sc-2025, Santa Cruz). ChIP was performed using a SimpleChIP Enzymatic Chromatin IP kit (Cat. #9003S, Cell Signaling Technology) as described previously (45). ChIP-ed DNA was quantified by qPCR using *ZTA*, EBV *RTA* primers, or KSHV *RTA* primers (63) as indicated (Table 2).

### Cell lysis, WB, and immunoprecipitation (IP)

Cell lysis, WB, and immunoprecipitation (IP) were performed as previously described (64), with minor modifications. The cells were harvested, lysed in 2 × SDS PAGE sample buffer, and boiled for 5 min. Proteins were separated on 4–20% TGX gels (Cat. #4561096; Bio-Rad), transferred to PVDF membranes, and probed with the indicated primary antibodies followed by horseradish peroxidase-conjugated secondary antibodies. See also Table 1 for antibody sources.

For IP, the cells were lysed on ice for 30 min in buffer containing 50 mM Tris-HCl (pH 7.5), 150 mM NaCl, 0.1% NP-40, and protease inhibitor cocktail (Cat. #4693116001; Sigma-Aldrich). Lysates were sonicated (10 s on/10 s off, three cycles, 35% amplitude) and clarified by centrifugation at 14,600 × *g* for 15 min at 4°C. Ten percent of the supernatant was reserved as input, and the remainder was incubated with the indicated magnetic beads. Input and IP-ed proteins were analyzed by WB using the specified antibodies.

### Flow cytometry

Approximately 5 × 10^6^ cells were harvested and maintained on ice, washed once with cold PBS, and pelleted by centrifugation at 1,200 × *g* for 5 min. The cells were resuspended in PBS and fixed with 4% paraformaldehyde for 10 min at room temperature, followed by two washes with PBS and resuspension in PBS. Immediately prior to data acquisition, samples were passed through a 40 µm cell strainer to remove aggregates. Fluorescence signals were acquired on a CytoFLEX 2-L flow cytometer and detected using a 525 nm filter (Beckman Coulter). Data were analyzed by CytExpert 2.6 software (Beckman Coulter).

### Lytic induction and EBV copy number detection

Akata (EBV+) cells were seeded at 1 × 10⁶ cells/mL in six-well plates. After 3 h, the cells were lytically induced by anti-human IgG (50 µg/mL; Cat. #0855087, MP Biomedicals) and harvested at the indicated time points as described previously (2, 45, 64). SNU-719 (EBV+) and HK-1 (EBV+) cells were seeded at 3 × 10⁵ cells/mL in six-well plates and incubated overnight. On the following day, SNU-719 cells were treated with TPA (20 ng/mL; Cat. #NC9325685, Fisher Scientific), while HK-1 cells were treated with a combination of TPA (40 ng/mL) and sodium butyrate (5 mM; Cat. #S0519, Tokyo Chemical Industry) to induce EBV lytic cycle.

To quantify EBV replication, intracellular EBV DNA and virion-associated DNA were measured by qPCR. For intracellular EBV DNA analysis, total genomic DNA was extracted using a genomic DNA purification kit (Cat. #A1120, Promega) according to the manufacturer’s instructions. Briefly, 2 × 10^6^ cells were harvested and resuspended in Nuclei Lysis Solution, followed by RNA removal by RNase A treatment. Proteins were precipitated with Protein Precipitation Solution, and the clarified supernatant was mixed with isopropanol to precipitate genomic DNA. The DNA pellet was washed with ethanol and resuspended in DNA Rehydration Solution. EBV DNA levels were quantified using primers targeting *BALF5* and normalized to *β-actin* genomic DNA.

Extracellular virion-associated DNA was extracted and quantified following established protocols (64, 65). Briefly, 120 µL EBV-containing culture supernatants were treated with RQ1 RNase-free DNase (Cat. #M6101; Promega) to remove free-floating DNA. The reaction was stopped using the supplied stop buffer, and then Proteinase K (Cat. #BIO-37084; Meridian Bioscience) and SDS were added to digest viral proteins and release virion-associated DNA. DNA was purified by phenol-chloroform extraction and precipitated with 250 μL of 100% isopropanol, 50 μL of 3M sodium acetate pH 5.2, and 1 μL glycogen at −80°C overnight. Pellets were washed with 70% ethanol, air-dried, and resuspended in Tris-EDTA (TE) buffer (10 mM Tris, 1 mM EDTA, pH 8.0). EBV DNA was quantified by qPCR using *BALF5*-specific primers (64).

### Ganciclovir treatment

Akata (EBV+) cells were pretreated with ganciclovir (GCV; 10 µg/mL) or solvent dimethyl sulfoxide (DMSO) as a control (33) for 1 h prior to anti-human IgG-mediated crosslinking of BCR to induce EBV lytic replication.

### RNA isolation, cDNA production, and RNA-seq

Total RNA was isolated using the ISOLATE II RNA Mini Kit (Meridian Bioscience) according to the manufacturer’s instructions. Briefly, cell pellets containing up to 5 × 10^6^ cells were lysed in RLY buffer supplemented with β-mercaptoethanol and homogenized by vortexing. Lysates were clarified using ISOLATE II filters, and RNA-binding conditions were adjusted by the addition of 70% ethanol. Samples were loaded onto silica spin columns, followed by membrane desalting and on-column DNase I treatment to remove genomic DNA. Columns were sequentially washed with RW1 and RW2 buffers, dried by centrifugation, and total RNA was eluted in RNase-free water. For gene expression analysis by qPCR, the cDNA synthesis was performed using High-Capacity cDNA Reverse Transcription Kit with RNase Inhibitor (Cat. #4374966, Applied Biosystems).

For RNA-seq, RNA concentration was measured using a Qubit 2.0 Fluorometer (Life Technologies, Carlsbad, CA, USA), and RNA integrity was assessed with an Agilent TapeStation 4200 (Agilent Technologies, Palo Alto, CA, USA). RNA-seq libraries were prepared using the NEBNext Ultra II RNA Library Prep Kit for Illumina according to the manufacturer’s instructions (NEB, Ipswich, MA, USA). Briefly, polyadenylated mRNA was enriched using oligo(dT) beads and subsequently fragmented for 15 min at 94°C. First- and second-strand cDNA synthesis was performed, followed by end repair and 3′ adenylation. Universal adapters were ligated to the cDNA fragments, and indexed libraries were generated by limited-cycle PCR amplification. Library quality was evaluated using the Agilent TapeStation, and library concentration was determined using a Qubit 2.0 Fluorometer (Invitrogen, Carlsbad, CA, USA) and qPCR (KAPA Biosystems, Wilmington, MA, USA).

Sequencing libraries were clustered on a flow cell and sequenced on an Illumina platform (HiSeq 4000 or equivalent) following the manufacturer’s protocols. Paired-end sequencing was performed with a read length of 2 × 150 bp. Image analysis and base calling were carried out using Illumina Control Software. Raw base call files were converted to FASTQ format and demultiplexed using bcl2fastq version 2.17, allowing one mismatch for index sequence identification.

### RNA-seq data analysis

RNA-seq data were processed using the Galaxy server (66). Raw FASTQ files were first assessed for quality using FastQC, followed by adapter and low-quality base trimming when necessary. Transcript abundance was quantified directly from trimmed reads using Salmon v.0.8.2 (67) in quasi-mapping mode against the Akata-EBV (KC207813.1) and human (GRCh38) combined transcriptome. Differential expression analysis was then performed using edgeR 3.34.0 + galaxy (68), including library size normalization, dispersion estimation, and statistical testing between experimental conditions. Genes with adjusted *P* values below 0.05 were defined as differentially expressed (see Table S2).

### *In situ* proximity ligation assay

Proximity ligation assay (PLA) was performed as previously described (64, 69). Briefly, Akata (EBV+) cells were blocked with 3% BSA in PBS for 1 h at room temperature, then incubated overnight at 4°C with either PBS control or a mixture of rabbit anti-HNRNPA2B1 (Cat. #14813-1-AP, Proteintech) and mouse anti-KDM1A (LSD1) (Cat. #67037-1-Ig, Proteintech), rabbit-anti RCOR1 (Cat. #27686-1-AP), and mouse anti-ZNF217 (Cat. #67730-1-Ig) antibodies (1:50 in 3% BSA). Probes were subsequently incubated at 37 °C for 1 h, followed by ligation and signal amplification using the NaveniFlex Cell Red Kit (Cat. #NC.MR.100, Navinci). Nuclei were counterstained with DAPI, and samples were imaged on a Nikon AXR confocal microscope.

### ChIP-seq analysis

ChIP-seq data sets were obtained from the NCBI Gene Expression Omnibus (GEO; accession GSE284859) (17) and processed using our previously established pipeline (69). Briefly, data sets were retrieved via the Galaxy web platform (66). Raw sequencing reads were first assessed for quality using FastQC, followed by trimming of low-quality bases and adapter sequences. High-quality reads were then aligned to both the human reference genome and the EBV Akata strain genome (GenBank: KC207813) using Bowtie2. Peak calling was performed with MACS2, using corresponding input DNA as a control to identify regions of significant enrichment (70). Processed alignment files and peak tracks were subsequently visualized using the Integrative Genomics Viewer (IGV) (71).

### Quantification and statistical analysis

Statistical analyses were performed using a two-tailed Student *t*-test with Microsoft Excel software. A *P* value less than 0.05 was considered statistically significant. The values are presented as means and standard deviations for biological replicate experiments as specified in the figure legends.

## Data Availability

The RNA-seq data sets have been deposited in the NCBI Gene Expression Omnibus (GEO) under accession number GSE320084. The proteomics data sets have been deposited in the ProteomeXchange Consortium via the PRIDE (72) partner repository with identifier PXD074669.

## SUPPLEMENTAL MATERIAL

Table S1 jvi.00613-26-s0001.xlsx Proteins identified by enChIP-MS at EBV ZTA promoter.

Table S2 jvi.00613-26-s0002.xlsx Differentially expressed genes in Akata (EBV+) cells expressing sg2-A2B1 versus sg-NC.

ASM does not own the copyrights to Supplemental Material that may be linked to, or accessed through, an article. The authors have granted ASM a non-exclusive, world-wide license to publish the Supplemental Material files. Please contact the corresponding author directly for reuse.
